# Metabolomic and Pharmacological Approaches for Exploring the Potential of *Tanacetum parthenium* L. Root Culture as a Source of Bioactive Phytochemicals

**DOI:** 10.3390/ijms26157209

**Published:** 2025-07-25

**Authors:** Aurelio Nieto-Trujillo, Rosendo Luria-Pérez, Francisco Cruz-Sosa, Carmen Zepeda-Gómez, María G. González-Pedroza, Cristina Burrola-Aguilar, Armando Sunny, José Correa-Basurto, José A. Guerrero-Analco, Juan L. Monribot-Villanueva, María Elena Estrada-Zúñiga

**Affiliations:** 1 Centro de Investigación en Recursos Bióticos, Facultad de Ciencias, Universidad Autónoma del Estado de México, Carretera Toluca-Ixtlahuaca km 14.5, San Cayetano, Toluca 50295, Mexico; anietot_ext@uaemex.mx (A.N.-T.); cba@uaemex.mx (C.B.-A.); 2 Unidad de Investigación en Enfermedades Oncológicas, Hospital Infantil de México Federico Gómez, Dr. Márquez No 162, Col. Doctores, Cuauhtémoc, Mexico City 06720, Mexico; rluria@himfg.edu.mx; 3 Departamento de Biotecnología, Universidad Autónoma Metropolitana-Unidad Iztapalapa, Av. Ferrocarril San Rafael Atlixco No 186, Leyes de Reforma 1ra Sección, Iztapalapa, Mexico City 09310, Mexico; cuhp@xanum.uam.mx; 4 Facultad de Ciencias, Universidad Autónoma del Estado de México, Campus El Cerrillo, Piedras Blancas, Carretera Toluca-Ixtlahuaca km. 15.5, Toluca 50200, Mexico; zepedac@uaemex.mx (C.Z.-G.); mggonzalezp@uaemex.mx (M.G.G.-P.); 5 Centro de Investigación en Ciencias Biológicas Aplicadas, Facultad de Ciencias, Universidad Autónoma del Estado de México, Carretera Toluca-Ixtlahuaca km 14.5, San Cayetano, Toluca 50295, Mexico; sunny.biologia@gmail.com; 6 Laboratorio de Modelado Molecular, Bioinformática y Diseño de Fármacos, Sección de Estudios de Posgrado e Investigación, Escuela Superior de Medicina, Instituto Politécnico Nacional, Plan de San Luis y Salvador Díaz Mirón S/N, Casco de Santo Tomás, Mexico City 11340, Mexico; corrjose@gmail.com; 7 Laboratorio de Química de Productos Naturales, Red de Estudios Moleculares Avanzados, Instituto de Ecología, A.C., Carretera Antigua a Coatepec 351, El Haya, Xalapa 91073, Mexico; joseantonio.guerrero@inecol.mx (J.A.G.-A.); juan.monribot@inecol.mx (J.L.M.-V.)

**Keywords:** antioxidant, antibacterial, cytotoxic, α-amylase inhibition, secondary metabolites, targeted metabolomics, untargeted metabolomics

## Abstract

*Tanacetum parthenium* (Asteraceae) has been traditionally used worldwide for medicinal purposes, and some of its therapeutic uses have been attributed to the pharmacological effects of its secondary metabolites. The root culture of this species might represent a sustainable source of several pharmacologically active compounds. The biomass of a root *T. parthenium* culture was extracted with methanol and fractionated using column chromatography. Three selected fractions (4TP, 5TP, and 8TP) were analyzed via spectrophotometric, chromatographic, and mass spectrometry techniques and in vitro pharmacological assays. The greatest values for total phenolic and phenolic acid contents and antibacterial activity against *Escherichia coli* were determined for 4TP. The highest values for total flavonoid and sesquiterpene lactone contents, antioxidant potential, and α-amylase inhibitory effect were determined for 8TP. The antibacterial effect against *Staphylococcus aureus* was not significantly different among the three fractions. The root culture of *T. parthenium* is a potential source of several metabolites, such as phenolic acids, fatty acids, coumarins, sesquiterpenoids, and triterpenoids, which are capable of exerting α-amylase inhibition and antioxidant, antibacterial, and cytotoxic effects. Among eight phenolic compounds detected and quantified in the fractions, chlorogenic acid was the most abundant.

## 1. Introduction

In vitro cultures of medicinal plant producers of secondary metabolites have gained significant attention in recent decades since they are a biotechnological tool representing a sustainable source of compounds able to exert pharmacological effects [1,2,3]. In vitro culture consists of several techniques for cultivating cells, tissues, organs, or plantlets under relative control of genetics, metabolism, and environmental growth conditions [1]. Among the applications of plant in vitro culture are the production of bioactive secondary metabolites because this method offers major advantages over whole plants, such as allowing uniform yield and quality of plant-derived products, shorter and continuous production cycles, efficient recovery of bioactive compounds, lack of environmental or genetic contamination due to controlled aseptic conditions, and scalable production from laboratory volumes to industrial bioreactors [4,5]. However, achieving industrial-scale production in a bioreactor implies great previous background research in flasks, laboratory volume bioreactors, and scale-up to industrial volumes, focused on determining the factors that influence biomass production and enhancing the yield production and recovery processes of specific secondary metabolites with pharmacological potential proven for designing drugs [6,7].

In recent years, the potential of in vitro cultures of medicinal plants as a source of biopharmaceutical compounds has been researched by determining the biological effects of their extracts via in vitro and in vivo pharmacological tests, e.g., antioxidant, antibacterial, cytotoxic, and antidiabetic effects [8]. Assessing the antioxidant capability of plant extracts has been widely reported since the overproduction of reactive oxygen species is responsible for the pathogenesis of many nontransmissible diseases, such as atherosclerosis, cancer, diabetes, inflammation, liver injury, aging, cardiovascular and neurodegenerative diseases, and others [9,10,11]. The antioxidant effect of these extracts has been related to phenolic compounds and terpenoid secondary metabolites because of their ability to scavenge free radicals [9,12,13]. Depending on their structure and chemical nature, these secondary metabolites might also exhibit other pharmacological activities, e.g., antibacterial, cytotoxic, and antidiabetic activities [9].

In addition, metabolomics can be a complementary tool for the plant in vitro cultures [14] to examine its potential to produce several pharmacologically active secondary metabolites. In recent decades, untargeted metabolomics has gained great importance in identifying the metabolites produced by organisms under specific conditions [15]. In medicinal plants, untargeted metabolomics can be applied to identify a vast number of secondary metabolites as a “fingerprint” related to pharmacological effects, which might also contribute to obtaining chemical quality control [15,16,17]. The putative identification of metabolites obtained during untargeted metabolomics may establish the basis for carrying out targeted metabolomics to confirm the identification and quantification of potential secondary biomarkers, such as those demonstrated in dark tea (*Camelia sinensis*) [18].

*Tanacetum parthenium* L. (Asteraceae) is an aromatic and perennial herb that occurs in the Caucasian region and southern Europe, but it was introduced and naturalized in other regions of Africa, Asia, and America [19]. This plant has been traditionally used worldwide for medicinal purposes, but it is strongly associated with inflammation and pain [20,21]. Several of the therapeutic properties reported for this species have been attributed to their secondary metabolites, as they have proven to have pharmacological effects; among those secondary metabolites, terpenes (sesquiterpene lactones and essential oils) and phenolic compounds (flavonoids and phenolic acids) are highlighted [20,21,22,23]. The sesquiterpene lactones reported for *T. parthenium* include nearly thirty compounds, such as artecanin, balchanin, canin, costunolide, parthenolide (PTN), reynosin, santamarine, and tanacetin, among others; PTN has been reported to be the most abundant [20] and the most pharmacologically important because of its significant anti-inflammatory, neuroprotective, cardioprotective, and anticancer effects [20,24,25]. While some flavonoids of this species, such as tanetin, quercetin, apigenin, luteolin, chrysoeriol, santin, and kaempferol derivatives, among others [20], have been found to be involved in anticancer, antioxidant, or anti-inflammatory activities [24,25]. Finally, essential oil has been reported to be responsible for antimicrobial activity, and some examples of this type of secondary metabolite reported in this species include camphor, farnesol, *trans*-chrysanthenyl acetate, camphene, borneol, *p*-cymene, and bornyl acetate; however, camphor has been reported as the most abundant compound [20,26,27,28,29].

Several works reporting *T. parthenium* in vitro cultures, whether focused on the propagation of this species or aimed at producing bioactive secondary metabolites, have focused mainly on PTN production because of its pharmacological effects, especially its anticancer activity [30,31,32,33,34,35,36,37,38]. In previous work, Nieto-Trujillo et al. [36] demonstrated that a *T. parthenium* root culture produced chlorogenic acid, caffeic acid, salicylic acid, and PTN, but their production depended on the time of culture; the largest amounts of all those secondary metabolites (1.347 ± 0.159 mg chlorogenic acid/g; 0.851 ± 0.099 mg caffeic acid/g; 0.19 ± 0.028 mg salicylic acid/g, and 0.0231 ± 0.011 mg PTN/g) were produced after thirty-two days of growth; however, in all those previous reports of this species, no attempts were made to assess pharmacological effects, or untargeted metabolomics for chemical profiling. Thus, to increase knowledge of our previous work, by Nieto-Trujillo et al. [36], we consider that combining these two methods could probably be useful for exploring the potential of a root culture of *T. parthenium* as a source of bioactive phytochemicals.

This work aimed to estimate the potential of *Tanacetum parthenium* root culture to produce several phytochemicals that have antioxidant, antibacterial, cytotoxic, and α-amylase effects in vitro through the phytochemical and pharmacological characterization of three fractions from a methanolic extract.

## 2. Results

### 2.1. Selection of Fractions

Phytochemical qualitative screening of phenolic compounds, flavonoids, and sesquiterpene lactones in a total of ten fractions obtained after the fractionation of the methanolic extract of *T. parthenium* root culture revealed a main intensity in precipitate positive response or color for three fractions, coded 4TP, 5TP, and 8TP; thus, these fractions were selected for further experiments.

### 2.2. The Three Fractions Exhibited Pharmacological Activity

**Figure 1:**
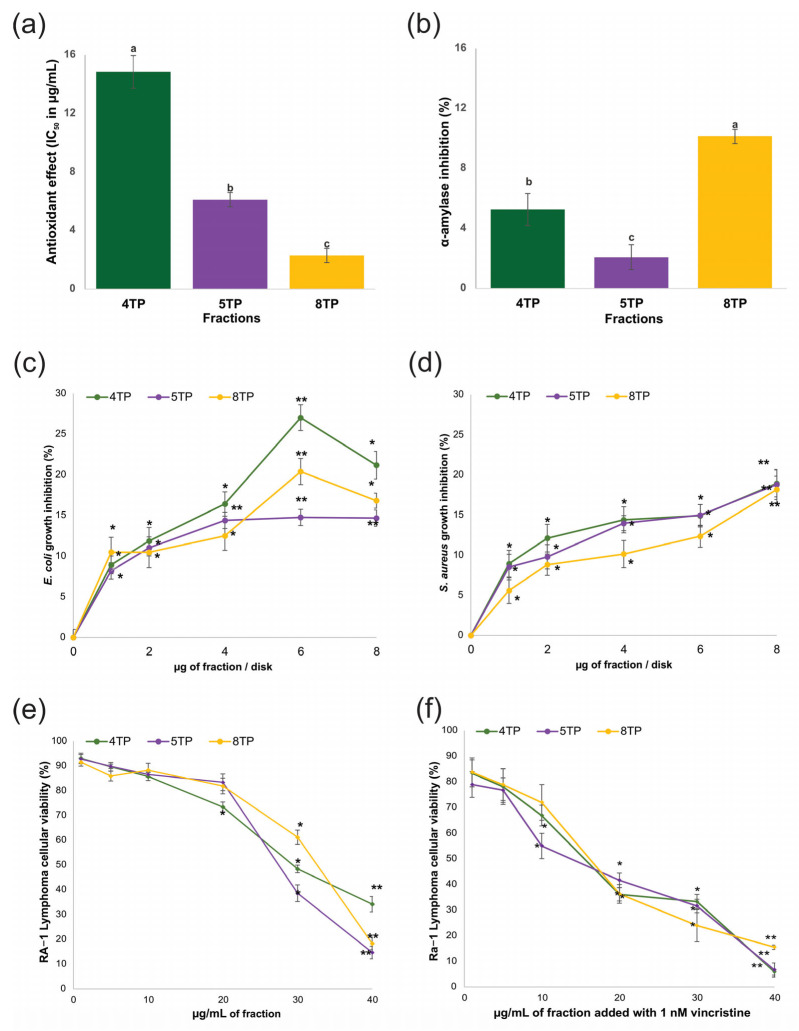
Pharmacological activities of the 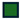 4TP, 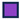 5TP, and 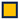 8TP fractions obtained from the methanolic extract of the root biomass of a *T. parthenium* culture. (**a**) Antioxidant, inhibitory effect of (**b**) α-amylase, on (**c**) *E. coli*, (**d**) *S. aureus*, cytotoxic effect against the Ramos RA-1 lymphoma cell line (**e**) alone and (**f**) combined with 1 nM vincristine. The data are presented as the means ± SD. In (**a**,**b**), the letters indicate significant differences at the 5% level of probability; from (**c**) to (**e**), * indicates significant differences at the 5% level of significance compared to the negative control, and ** indicates the treatments showing the lowest values at the 5% level of significance compared to the negative control. The corresponding controls tested for those assays were as follows: (**a**) MeOH could not scavenge DPPH radical; (**b**) the positive control (acarbose) had an IC_50_ of 2.48 ± 0.31 μg/mL; (**c**,**d**) the negative control for both antibacterial assays was 3% DMSO, while the positive control for *E. coli* was 1 μg chloramphenicol/disk and the positive control for *S. aureus* was 1 μg vancomycin/disk; and (**e**) the negative control was the culture medium (which induced a cellular viability of 94.71% ± 1.5%), while 1 nM vincristine was used as a positive control (reducing cellular viability at 72.61% ± 1.45%).

The 4TP, 5TP, and 8TP fractions significantly affected the antioxidant, antibacterial, cytotoxic activities, and inhibition of α-amylase enzyme, generally depending on the tested concentration and type of fraction (Figure 1a–f). Significant differences in antioxidant and α-amylase inhibitory effects between the three fractions were determined, with the greatest values occurring for both effects in the 8TP fraction (IC_50_ of 2.3 μg/mL for scavenging the DPPH radical; 10.15% enzyme inhibition at a concentration of 10 µg/mL) (Figure 1a,b). The control tested for the antioxidant assay (MeOH) could not scavenge the DPPH radical. When the results of the enzyme inhibition were expressed as mg of acarbose equivalents per gram of fraction (mg AE/gF), 8TP had the highest value (23.36 ± 3.3 mg AE/gF) compared with 4TP and 5TP (12.12 ± 3.27 and 4.80 ± 1.73 mg AE/gF, respectively). A comparison between the 8TP fraction and the positive control (acarbose) regarding the effect of α-amylase inhibition revealed that the fraction with a significantly lower percentage of inhibition than that observed in the positive control (IC_50_ of 2.48 ± 0.31 μg acarbose/mL). All fractions significantly caused the inhibition of the growth of both bacteria at all the tested concentrations, but the highest percentage of bacteria inhibited against *E. coli* was found at 6 μg/disk, which was significantly different among the fractions. Thus, the percentage of bacteria inhibited at 6 μg/disk concentration decreased in the order 4TP > 8TP > 5TP (corresponding to 27.0%, 20.4%, and 14.8%, respectively) (Figure 1c). For *S. aureus*, the highest inhibition percentage for any fraction was reached at 8 μg/disk, and the three fractions were not significantly different (for 18.80–18.93%) (Figure 1d). Since the percentage inhibition against these bacteria was estimated for the positive control (antibiotic), the results showed that the fractions possessed a lower antibacterial effect than the antibiotic drug reference. Finally, for all the fractions, the cellular viability of the Ramos RA-1 lymphoma cells significantly decreased at concentrations higher than 20 μg/mL, with the best cytotoxic effect found at 40 μg/mL (cellular viability of approximately 35% for 4TP and 15% for 5TP and 8TP) (Figure 1e). A cytotoxic activity comparison between fractions determined the 5TP fraction as the best (IC_50_ of 26.37 ± 1.31 μg/mL against 30.91 ± 0.76 and 30.30 ± 0.94 μg/mL, for 4TP and 8TP, respectively). By contrasting the cytotoxic effect of the fractions with that of the positive control (1 nM vincristine), the fractions were found to be more toxic since this drug reference caused a cellular viability of 72.61% ± 1.45%. Moreover, the combination of each fraction at different concentrations with 1 nM vincristine caused enhanced cytotoxicity on the cells compared to the results obtained for the fractions alone; this effect depended on the type of fraction and its concentration (Figure 1f). The best cytotoxic effect of the combination of the fraction and vincristine mixture occurred for 4TP and 5TP at 40 μg/mL, which caused almost 5% cellular viability (Figure 1e). A comparison of the IC_50_ values for each fraction combined with 1 nM vincristine showed no significant differences (17.88 ± 1.41, 16.71 ± 1.26, and 18.45 ± 1.99 μg/mL for 4TP, 5TP, and 8TP with 1 nM vincristine, respectively).

### 2.3. Phytochemical Characterization of the Three Fractions

#### 2.3.1. Total Secondary Metabolite Contents Profile of Fractions

**Figure 2:**
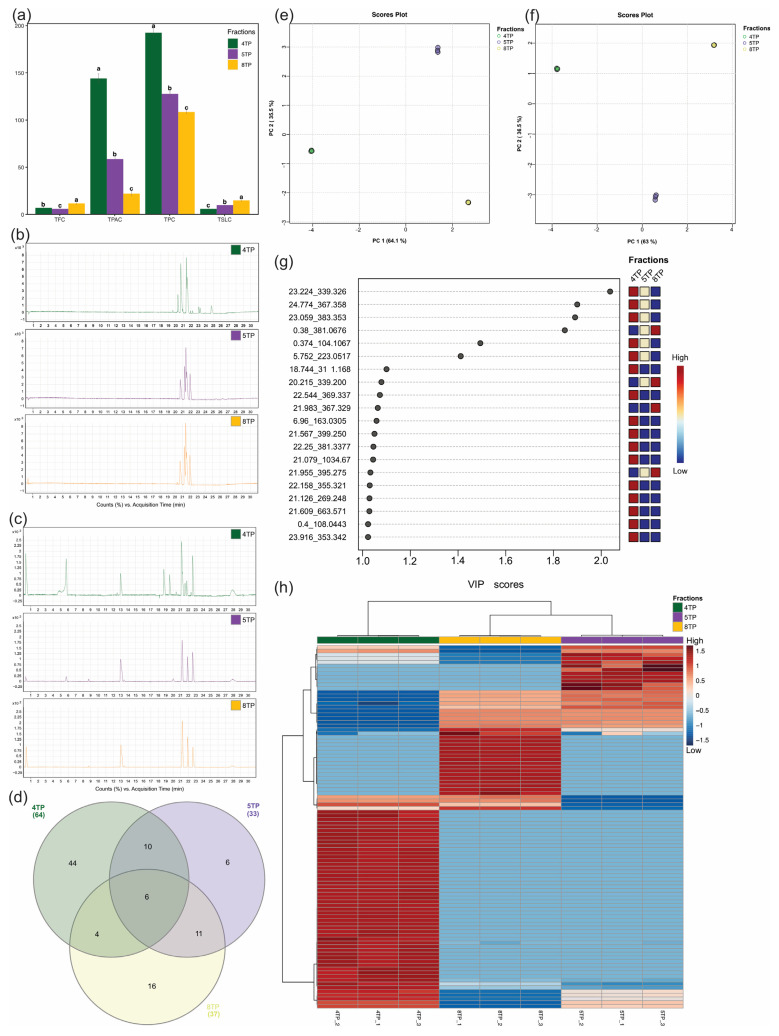
Differential compounds identified between the 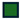 4TP, 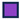 5TP, and 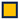 8TP fractions obtained from the methanolic extract of the root biomass of the *T. parthenium* culture. (**a**) Total secondary metabolite contents: phenolic, phenolic acid, flavonoid, and sesquiterpene lactone (TPC, TPAC, TFC, and TSLC, respectively); LC-MS chromatogram obtained via electrospray ionization in (**b**) negative ionization mode and (**c**) positive ionization mode; (**d**) Venn diagram; (**e**) PCA; (**f**) PSL-DA; (**g**) VIP score of the top 20 compounds; and (**h**) heatmap of all identified compounds. In (**a**), data show the means ± SD; letters indicate significant differences at the 5% probability level, and the results for TPC, TPAC, TFC, and TSLC are expressed as milligrams of gallic acid, verbascoside, quercetin, or parthenolide equivalents per gram of fraction, respectively (mg GAE/gF, mg VBE/gF, mg QE/gF, or mg PTNE/gF). The Venn diagram in (**d**) was constructed online at http://www.interactivenn.net/, accessed on 20 August 2024. Data from (**e**) to (**h**) were analyzed for the rt_m/z signal and its abundance (peak intensity) obtained from LC-MS results.

The phytochemical characterization of the 4TP, 5TP, and 8TP fractions, estimated by determining the total secondary metabolite contents (TPC, TPAC, TFC and TSLC), revealed significant differences (Figure 2a). The 4TP fraction had the highest values for TPC and TPAC (194.2 mg GAE/gF and 143.6 mg VBE/gF, respectively; Figure 2a), while the 8TP fraction had the highest values for TFC and TSLC (11.9 mg QE/gF and 14.7 mg PTNE/gF, respectively; Figure 2a).

#### 2.3.2. Differential Abundance of Compounds Between the Three Fractions from Untargeted Metabolomics

After processing the chromatographic data obtained from the LC-MS analysis by the Molecular Feature Extraction algorithm, based on spectrometric features (retention time_m/z ratio values) of the chromatographic, 97 compounds were distributed among the 4TP, 5TP, and 8TP fractions. The three fractions exhibited different chromatographic profiles, with several notable chromatographic peaks eluted from 20.5 to 23 min of retention time in the negative (Figure 2b) and positive (Figure 2c) ionization modes by electrospray. The Venn diagram showed a differential distribution of those compounds between fractions; 64, 33, and 37 signals were detected in the 4TP, 5TP, and 8TP fractions, respectively, with 44, 6, and 16 signals observed solely in the 4TP, 5TP, and 8TP fractions, respectively; at the same time, six signals were found in common in the three fractions (Figure 2d). The PCA, which was used to determine the relationship of the compound abundances between the three fraction replicates, indicated high (64.1%) variation (Figure 2e), which was also confirmed with PLS-DA (63%; Figure 2f), the VIP score (Figure 2g), and a heatmap (Figure 2h).

#### 2.3.3. The Phytochemical and Pharmacological Activities and Compound Abundances of the Three Fractions Were Correlated

**Figure 3:**
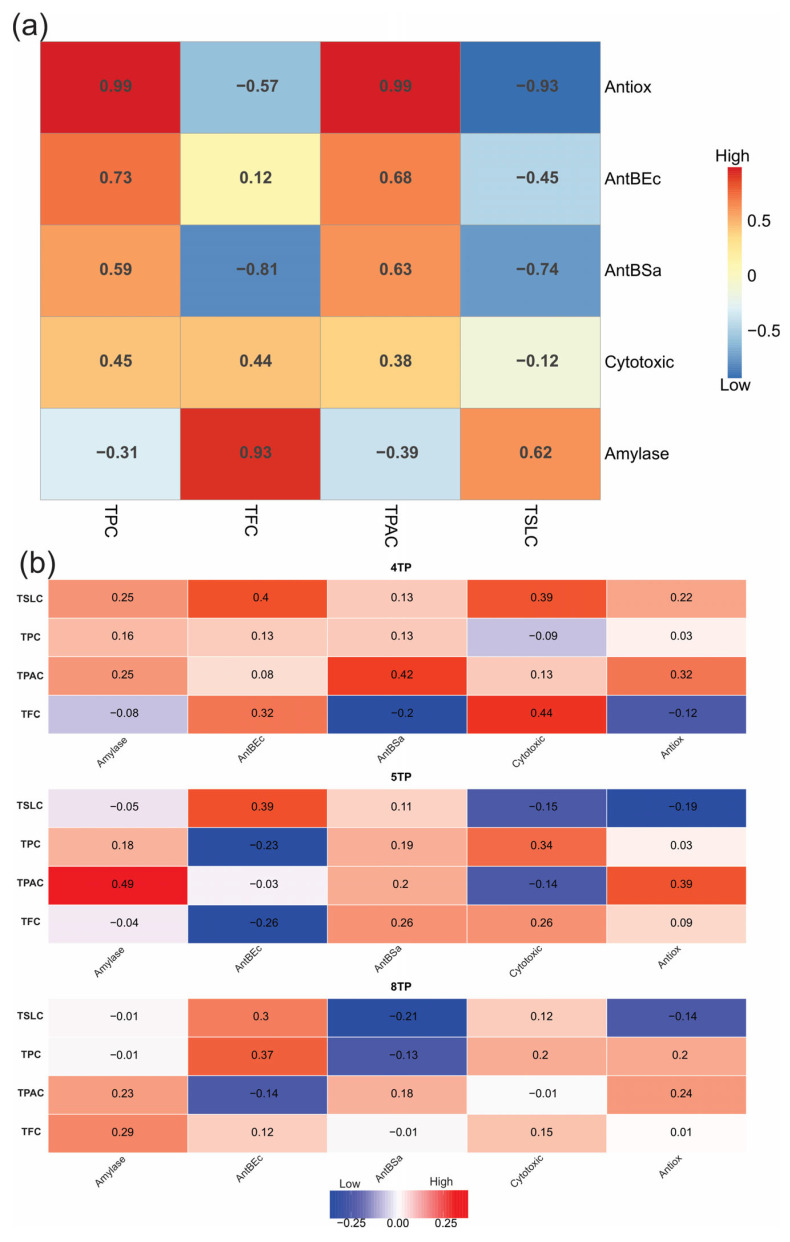

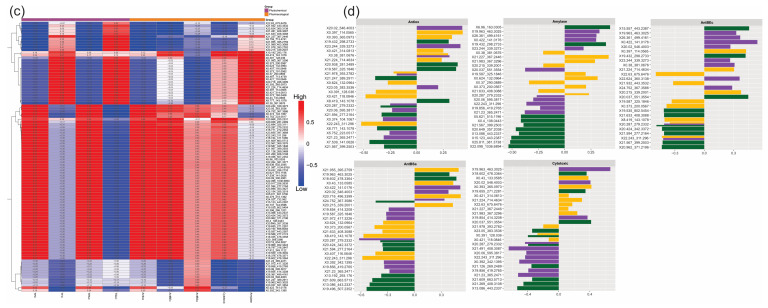
Correlations between total secondary metabolite contents (TPC, TPAC, TFC, and TSLC), pharmacological activities (antioxidant, antibacterial, cytotoxic, and α-amylase inhibition), and compound abundances among the 4TP, 5TP, and 8TP fractions obtained from the methanolic extract of the *T. parthenium* root culture biomass. Heatmap showing correlation values between pharmacological activities and total secondary metabolite content (**a**) among all three fractions and (**b**) by every fraction, (**c**) heatmap showing correlation values between compound abundance and pharmacological activities and total secondary metabolite content in the three fractions, and (**d**) heatmap showing correlation values between compound abundance and pharmacological activities in every fraction (showing the top correlations for 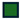 4TP, 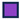 5TP, and 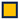 8TP). Values ranging from −1 to 1 represent the value of each correlation.

The phytochemical profiles (TPC, TPAC, TFC, and TSLC) of the three fractions were correlated with their pharmacological effects; these correlations and their types (positive or negative) were significantly influenced by the type of secondary metabolite (Figure 3a). TPC and TPAC were positively correlated with antioxidant, antibacterial, and cytotoxic effects in the following order from high to moderate: antioxidant > antibacterial against *E. coli* > antibacterial against *S. aureus* > cytotoxic (Figure 3a). TFC was significantly correlated with α-amylase inhibition and cytotoxic effects in the following order from high to moderate: α-amylase inhibition > cytotoxic (Figure 3a). Moreover, the TSLC was significantly correlated, in a moderate way, with the α-amylase inhibition effect (Figure 3a). When every fraction was correlated with the phytochemical profile and pharmacological effects, more moderate positive correlations were observed in the 4TP fraction than in the 5TP and 8TP fractions (Figure 3b). In addition, approximately 30% of the total 97 compounds showed a positive correlation with TFC, TSLC, α-amylase inhibition, and cytotoxic effects. Nearly 60% also had a positive correlation with TPC; TPAC; and antioxidant, antibacterial, and cytotoxic effects, and the remaining percentage was not correlated with any total secondary metabolite content but was correlated with antibacterial effects against *S. aureus* (Figure 3c). When the abundances of the top compounds were correlated with every pharmacological effect or total secondary metabolite content and compared among the fractions, it was observed that the fractions had different compounds able to exert particular pharmacological effects and even the same compound contributing to different pharmacological effects (Figure 3d); thus, the compounds showing the greatest values were selected for putative identification.

#### 2.3.4. Putative Identification of Metabolites Correlated with Pharmacological Effects

**Figure 4:**
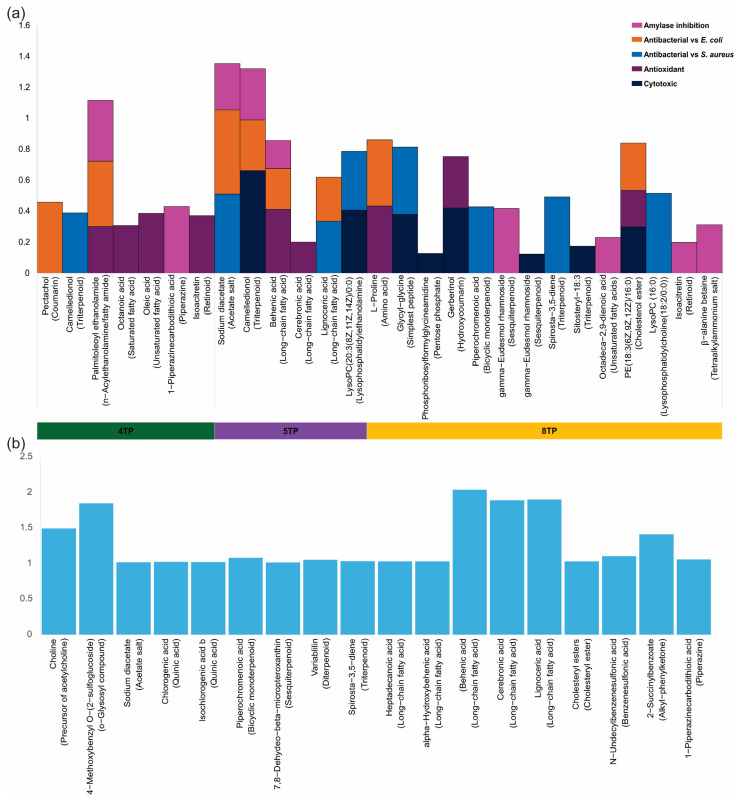
Compounds from the 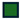 4TP, 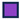 5TP, and 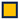 8TP fractions obtained from the methanolic extract of the root biomass of the *T. parthenium* culture correlated with pharmacological effects, and those compounds were determined mainly by the VIP score. (**a**) Putatively identified compounds in the 4TP, 5TP, and 8TP fractions showing correlation values related to pharmacological effects; (**b**) putatively identified compounds showing the main values of the VIP score.

According to the putative identification, some of the compounds that predominantly contributed to the pharmacological effects of the 4TP and 5TP fractions were fatty acids and derivatives (octanoic acid, oleic acid, palmitoleoyl ethanolamide, behenic acid, lignoceric acid, and cerebronic acid), triterpenoid (camelledionol), retinoid (isoacritretin), piperazine (1-piperazinecarbodithioic acid), coumarin (pectachol), acetate salt (sodium diacetate), and lysophosphatidylethanolamine. In contrast, in the 8TP fraction, the compounds contributing to pharmacological effects were mostly amino acids and derivatives (L-proline, glycyl-glycine, and β-alanine betaine), retinoid (isoacritretin), bicyclic monoterpenoid (piperochromenoic acid), sesquiterpenoid (gamma-eudesmol rhamnoside), triterpenoid (spirosta-3,5-diene), hydroxy coumarin (gerberinol), cholesterol ester and lysophosphatidylcholine (Figure 4a, Table S1, and Figure S1). In addition, the putative identification of the compounds that exhibited the highest VIP scores from the PLS-DA showed that at least seven compounds, fatty acids (behenic acid, lignoceric acid, and cerebronic acid), bicyclic monoterpenoid (piperochromenoic acid), piperazine (1-piperazinecarbodithioic acid), triterpenoid (spirosta-3,5-diene), and acetate salt (sodium diacetate), were correlated with the pharmacological effects of the fractions (Figure 4a,b, Tables S1 and S2, and Figure S1). The other compounds identified among those found in the PLS-DA to have the highest VIP scores were choline, 2-succinylbenzoate, variabilin, chlorogenic acid and isochlorogenic acid b, among others (Figure 4b, Table S2, and Figure S1).

#### 2.3.5. Targeted Metabolomics in Fractions

**Figure 5:**
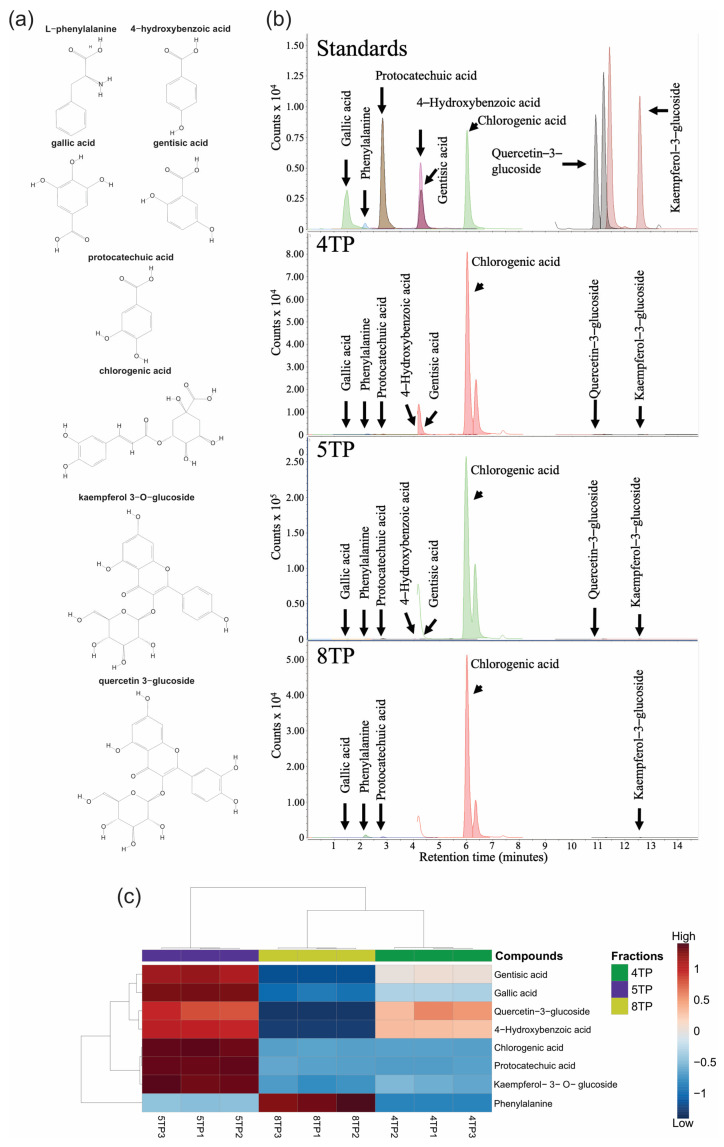
Compounds were detected and quantified through targeted metabolomics from the 4TP, 5TP, and 8TP fractions obtained from the methanolic extract of the root biomass of the *T. parthenium* culture. (**a**) The chemical structures of amino acid (L-phenylalanine), hydroxybenzoic acids (4-hydroxybenzoic acid, gallic acid, gentisic acid, and protocatechuic acid), caffeoylquinic acid (chlorogenic acid), and flavonoids (kaempferol 3-O-glucoside and quercetin 3-glucoside); (**b**) chromatogram and (**c**) heatmap of the concentrations of phenolic compounds identified through targeted metabolomics in the 4TP, 5TP, and 8TP fractions. The chemical structure images in (**a**) were obtained from the PubChem database (https://pubchem.ncbi.nlm.nih.gov/, accessed on 15 October 2024); in (**c**), the Euclidan distance and Ward algorithm were used for sample clustering.

Based on the untargeted analysis, phenolics targeted metabolomics allowed for the identification of a total of four hydroxybenzoic acids (4-hydroxybenzoic acid, gallic acid, gentisic acid, and protocatechuic acid), one caffeoylquinic acid (chlorogenic acid), two flavonoids (kaempferol 3-O-glucoside and quercetin 3-glucoside) and L-phenylalanine amino acid (Figure 5a), which were detected and quantified in the 4TP, 5TP, and 8TP fractions (Figure 5b and Table S3). Some compounds showed concentration differences among the three fractions (Figure 5c). However, the most abundant compound was chlorogenic acid, and the concentration was greater in the 5TP fraction (37,902.8 µg/gF) than in the 4TP and 8TP fractions (4204.7 and 4119.2 µg/gF, respectively); the rest of the compounds were detected at low concentration in the three fractions, except for phenylalanine, which was detected at concentrations of 119.14, 22.64 and 3.22 µg/gF for 8TP, 5TP and 4TP, respectively (Table S3). According to the correlations between phytochemical and pharmacological effects and compound abundances (Figure 3c), chlorogenic acid (rt_m/z of 23.916_353.3427) abundance was strongly positively correlated with the TPC, TPAC, IC_50_, and antibacterial effect against *E. coli* (0.97, 0.96, 0.96 and 0.85, respectively), and it was moderately positively correlated with the cytotoxic effect (0.61) and antibacterial effect against *S. aureus* (0.44) (Figure 3c).

## 3. Discussion

### 3.1. Fractions Obtained from Tanacetum parthenium Root Culture Showed Increased Total Secondary Metabolite Contents Regarding Crude Extracts

The results obtained in this work highlight that the root culture of *T. parthenium* represents a source of several secondary metabolites, exhibiting that a wide range of pharmacological activities and combining conventional and cutting-edge approaches such as untargeted metabolomics allowed for the identification of some of the phenolic compounds and terpenoids responsible for those effects. Although targeted metabolomics contributed to confirming the putative identification of these compounds, most focused on phenolic compounds because they exhibited high values in phytochemical analysis, and showed pharmacological effects (Table 1). Several reports on tissue cultures of *T. parthenium* have been published on targeted metabolomics for PTN since its production is influenced by genotype, type of organ (higher in aerial parts), developmental stage, cultivation, and environmental factors [32,39,40,41]. Several reports of the in vitro cultures of this species have demonstrated that PTN production depends on the type of culture (higher in organ cultures than in cell cultures), carbon source, and plant growth regulator (PGR) [34,42]. Other reports have focused on detecting other secondary metabolites, such as those in hairy roots culture, where the production of spiroketalenolethers [32] and PTN [31] was described. In callus cultures, phenolic compounds (scopoletin and isofraxidin coumarins) [33] or terpenoids, such as PTN [32,34], sitosterol and stigmasterol [30], and germacrene D [31], were reported; however, in those works, pharmacological assays and untargeted metabolomics, were not carried out, limiting the understanding of the full pharmacological potential and the identification of unknown bioactive compounds.

Moreover, testing fractions from a raw extract for pharmacological effects implemented in this work increases the total secondary metabolite content, which enhances pharmacological effects compared to previous reports about whole plants or tissue cultures. In this work, the highest values determined for total secondary metabolites were as follows: TPC, 194.2 mg GAE/gF; TPAC, 143.6 mg VBE/g; TFC, 11.9 mg QE/gF; and TSLC, 14.7 mg PTNE/g. For instance, in *T. parthenium* grown in a greenhouse and open field, under adequate-water conditions, the TPC (64.7 and 110.5 mg GAE/g, respectively) in the aerial parts was greater than that observed under reduced watering (33.45 and 93.85 mg GAE/g, respectively) [39]. The amount of TPC reported in the essential oil of *T. parthenium* was 152.2 mg GAE/g, while the TFC was 70.2 mg QE/g [43]. An 80% alcohol extract of the golden variety of *T. parthenium* had a TPC of 21.21 mg GAE/g, which was higher than that reported for *T. vulgare*; this extract showed a strong antioxidant effect and was potentially attributed to luteolin; PTN [44]; and caffeoylquinic acids, such as 3,5-dicaffeoylquinic acid, 3,4-dicaffeoyl-quinic acid, and 4,5-dicaffeoyl-quinic acid (comprising isochlorogenic acid A, B and C, respectively) [44]. The TPC of the shoots and roots of the in vitro plantlets changed after 32 days of culture, and it was enhanced when the plantlets were grown in PGR-enriched culture media (0.27 µM α-naphtalenacetic acid with 2.32 µM kinetin); the greatest TPC (62.54 mg GAE/g) was observed in the shoots from the plantlets treated with PGR-enriched culture for 10 days [35]. In a root culture, the TPC also changed after 32 days of culture, and the highest value (42.52 mg GAE/g) was determined at the end of the experiment (32 days) [35]. The PTN concentration in the plant material of *T. parthenium* maintained in vitro was 5 × 10^−3^% in long-term cell culture, while in leaves from regenerated cultured shoots, it was 1.08%; moreover, in glasshouse-grown plant material, it was 2.77% in apical leaves, 2.3% in mature flowerheads, and 6.5 × 10^−3^% in roots [32]. The hydroethanolic extract from the different organs (aerial parts, flower heads, and leaves) of *T. parthenium* had different phytochemical profiles. The highest total content of several secondary metabolites, such as polyphenols (67.41 mg GAE/mL), flavonoids (19.33 mg catechin equivalent/mL), and phenolic acids (5.10 mg caffeic acid equivalent/mL), as well as the highest antioxidant potential, were observed in the flowers. In contrast, the extract of aerial parts had the highest content of condensed tannins (22.89 mg delphinidin/mL) and terpenoids (54.41 mg linalool/mL), and also exhibited the most effective antimicrobial activity [45]. In addition, an HPLC analysis showed that caffeoylquinic acids (e.g., chlorogenic acid at 3.98 mg/mL; isochlorogenic acids A, B, and C) were present at higher concentrations (5.33, 1.28 and 0.59 mg/mL, respectively) than flavonoids (apigenin, quercetin, santin, and kaempferol-3-rutinoside at 0.05, 0.06, 0.32, and 0.4 mg/mL, respectively) [45].

### 3.2. Fractions Obtained from Tanacetum parthenium Root Culture Exhibited Enhanced Pharmacological Activity as Compared to Crude Extracts

Regarding the antioxidant effect, in the present study, IC_50_ of 2.3, 6.11, and 14.84 µg/mL were achieved for the 8TP, 5TP and 4TP fractions, respectively, indicating a better activity than that reported for the extracts. For instance, an IC_50_ of 33.22 µg/mL has been reported for the 70% methanol extract of *T. parthenium* using the radical DPPH, from which phenolic acids (ferulic acid) and flavonoids (apigenin, luteolin, luteolin-7-O-glucoside, kaempferol, and chrysophanol) were isolated and determined to be potent antioxidant agents (IC_50_ values from 6.44-16.23 µM) based on the DPPH assay [46]. The essential oil of *T. parthenium* had an IC_50_ of 30.23 µg/mL according to the DPPH assay; it was suggested that the high DPPH scavenging activity was attributed to the content of monoterpenes and sesquiterpene alcohols, e.g., camphor (the most abundant at 43.97%), chrysanthenyl acetate, germacrene D and B, and farnesol [43]. The 80% alcohol extract of the golden variety of this species showed a strong antioxidant effect (84% of DPPH free radical-scavenging activity) that was potentially attributed to luteolin; PTN [44]; and caffeoylquinic acids, such as isochlorogenic acid B and C [44]. The superior antioxidant capacity of the fractions examined in this study highlights their relevance for future applications.

The α-amylase inhibitory effect of extracts obtained from species belonging to the *Tanacetum* genus has been reported. For example, the methanol and ethyl acetate extracts of different organs (leaf + stem, capitulate, and herb) of *T. haussknechtii* showed from 263.3 to 320.7 mg of acarbose equivalents per gram of extract (mg AE/g) in the methanolic extract, and 258.8 to 356.9 mg AE/g in the ethyl acetate extract. The greatest inhibitory effect (356.9 mg AE/g) determined in capitula was attributed to caffeoylquinic acid and flavonoids, which are reported to have antidiabetic effects [47]. Similarly, the type of extract obtained from the aerial parts of *T. poteriifolium* influenced α-amylase inhibition, with the greatest inhibitory effect (0.7 mmol AE/g ~451.9 mg AE/g) observed in the ethyl acetate extract compared to that in the MeOH (0.51 mmol AE/g ~322.8 mg AE/g) and water (0.10 mmol AE/g ~64.5 mg AE/g) extracts; this inhibitory effect was correlated with TFC (0.91), although this effect was observed as poor [48]. The type of organ (flower head, aerial parts and roots) of *T. macrophyllum* and *T. speciosa* influenced the inhibitory effect of the 80% MeOH extract on α-amylase; in both species, the greatest inhibitory effect was detected for flower heads (0.65 mmol AE/g ~419.6 mg AE/g for *T. macrophyllum*, and 0.58 mmol AE/g ~374.4 mg AE/g for *T. speciosa*) [49]. Additionally, the type of organ (flowers, stems, and aerial parts) of *T. vulgare* and its extracts (prepared with hexane, EtOH/water, and infusion) influenced α-amylase inhibition and the highest effect was detected in the hexane extracts of flowers and stems (0.53 and 0.50 mmol AE/g ~342.1 and 322.8 mg AE/g; the authors argued that this effect might be attributed to phenolics and sesquiterpene lactones [50]. Finally, the inhibition of α-amylase was not affected by the extraction method (maceration, Soxhlet extraction, ultrasound, microwave extraction, and accelerated extraction) with ethanol used in the aerial parts of *T. parthenium*; the inhibitory effect ranged from 0.51 to 0.56 mmol AE/g ~329.2 to 361.5 mg AE/g [48]. In the present study, the enzyme inhibition levels shown by all the fractions were 23.36, 12.12, and 4.80 mg AE/gF for 8TP, 4TP, and 5TP, respectively, which are capable of inhibiting the enzyme at 10.15, 5.27, and 2.09% at 10 μg/mL, respectively; these values were lower than the IC_50_ of acarbose (2.48 ± 0.31 μg/mL). However, in previous reports, the α-amylase inhibition activity of the samples was expressed as the acarbose equivalent per gram, but data about that percentage were not shown. Enzyme inhibition is considered a therapeutic strategy for managing type II diabetes, where α-amylase is a blank target because it is related to the breakdown of sugars in the intestine [48].

Moreover, *T. parthenium* has been proven to have antibacterial effects against *E. coli* and *S. aureus*, which was also observed in the present work, where the fractions of methanolic extract provoked the highest inhibition of 27.02% in *E. coli* after treatment with fraction 4TP at 6 µg per disk and approximately 18% in *S. aureus* after the exposure to the three fractions tested at 8 µg per disk. While the essential oil of *T. parthenium* has a proven antibacterial effect, it had different results among the types of bacteria tested. An inhibition rate of 89.44% was shown for *S. aureus* (at a 20 µL dilution with ethanol at a 1:5 ratio, applied per disk), while against *E. coli*, no inhibition was observed [26]; the authors attributed this effect to the presence of camphor. Similarly, Ghavam [30] reported an inhibition rate of 38.8% in *S. aureus* with 10 µL of essential oil per well, but against *E. coli*, there was no inhibition effect. In another study, Shafaghat et al. [29] demonstrated that the essential oil of *T. parthenium*, at 30 µL per disk, inhibited the growth of *E. coli* by 68.10%, while for *S. aureus*, it inhibited growth by 89.44%, and the authors attributed the effect to the presence of the oxygenated compounds. Izadi et al. [28] reported 64.91% growth inhibition for *S. aureus*, 68.42% growth inhibition for *E. coli* ATCC 25923, and 74.46% growth inhibition for *E. coli* ATCC 157 using essential oil at 2.5 µL per disk. Polatoğlu et al. [51] reported a minimum inhibitory concentration (MIC) of 125 µg essential oil/mL for *E. coli* and *S. aureus*.

Several species of the *Tanacetum* genus have demonstrated cytotoxic effects on cancer cell lines. The methanol extracts of *T. macrophyllum*, *T. vulgare*, and *T. corymbosum* inhibited cell growth by 69.87, 77.68, and 93.71%, respectively, at 200 µg/mL in HeLa cells (human cervical cancer) after 24 h of exposure [52]. The IC_50_ of the *T. erzincanense* methanol extract in the MCF-7 (breast adenocarcinoma), HepG2 (hepatocellular carcinoma), and HT-29 (human colorectal adenocarcinoma) cell lines were 41.3, 46.9, and 63.8 µg/mL, respectively, after 48 h of exposure; in this extract the TPC was 64.4 mg GAE/g and the TFC was 62.2 mg QE/g; and both had high positive correlations (0.795 and 0.671, respectively) with cytotoxic activity [53]. The IC_50_ of the *T. vulgare* leaf methanol extract in the HeLa cell line after 72 h of exposure was approximately 50 µg/mL; this cytotoxic activity was attributed to its phenolic acid content [54]. The IC_50_ for PTN after 48 h of exposure was reported to be 1.26 µg/mL for the Raji cell line (Epstein–Barr virus-positive lymphoma) [55], 2.1 µg/mL for SiHa (human cervical cancer) cells, and 2.4 µg/mL for MCF-7 cells. Another study determined an IC_50_ of 5 µg/mL after 72 h of exposure to PTN in the MT2 cell line (lymphoma cells infected with human T-cell leukemia virus type 1) [56]. In the present study, the three fractions showed cytotoxic effects on the RA-1 cell line (human B non-Hodgkin lymphoma), with IC_50_ values of 26.37, 30.91 and 30.30 µg/mL for 5TP, 4TP and 8TP, respectively. These data revealed that the fractions obtained in this work had better cytotoxic effects than the complete extracts of other species of the *Tanacetum* genus; however, compared to those of PTN, the fractions obtained in this study had lower cytotoxic activity. In addition, the results of the present work, in which the fractions were combined with 1 nM vincristine (added 6 h before completing the 24 h incubation) might cause chemosensitization because the cytotoxic effect of the fractions alone was enhanced, provoking IC_50_ values of 16.71, 17.88 and 18.45 µg/mL, for 5TP, 4TP, and 8TP, respectively. Chemosensitizing can occur due to the synergistic or additive effect of the secondary metabolite profile, which can be greater than the sum of the individual actions given by different individual action mechanisms [57].

### 3.3. Correlations Among Pharmacological Activities, Phytochemical Profiles, and Untargeted Metabolomics of Fractions Enabled the Identification of Metabolites

For this work, determining the correlation among pharmacological activities, phytochemical characterization, and untargeted metabolomics proved useful for identifying secondary metabolites, such as phenolic compounds, mainly phenolic acids and terpenoids, that are strongly associated with the pharmacological activity of the three fractions. Additionally, this approach allows for the detection of other metabolites potentially participating in biological effects, such as fatty acids (Table 1, Tables S1 and S2). Although *T. parthenium* has been described as a promising source of bioactive phytochemicals related to its medicinal uses, its chemical composition is limited [58]. Recent studies have reported large amounts of succinic, palmitic, linoleic, and linolenic acids (4.89, 2.15, 1.71, and 1.07 mg/g, respectively) and benzoic acid (1.89%) [58]. Furthermore, Rezaei et al. [44] reported thirteen fatty acids, such as palmitic acid (the most abundant at 57.27%), myristic acid, lauric acid, capric acid, palmitoleic acid, oleic acid, linoleic acid, linolenic acid, arachidic acid, behenic acid, erucic acid, and lignoceric acid, in the essential oil of *T. parthenium*. This species has also been recognized as a source of sesquiterpenes, monoterpenes, flavonoid glycosides (e.g., quercetin, 6-hydroxykaempferol 3,6-dimethyl ether, and 6-methoxykaempferol 3-methyl ether), triterpenoids (e.g., oleanolic acid methyl ester), phytosterols closely resembling cholesterol (e.g., campesterol, fucosterol, β-sitosterol, and stigmasterol), fatty acids (e.g., palmitic, myristic, lauric, linoleic, and capric acids), phenolic acids (e.g., isochlorogenic, chlorogenic, syringic, ferulic, sinapic, vanillic, and *p*-coumaric acids), coumarins (e.g., isofraxidin and 9-epipectachol B), and pyrethrin, among others [20]. In this work, several of those metabolites were identified (Table 1, Tables S1 and S2), and they have been credited in the literature for their biological effects (Table 1), supporting the results observed through the analysis carried out in this research. In addition, the metabolites that exhibited the highest VIP scores from the PLS-DA could be used as chemical biomarkers of the fractions, especially the compounds that were also correlated with pharmacological effects, such as chlorogenic acid, which might be useful for quality control for studying and developing drugs, nutraceuticals, or standardized extracts, among others, from the three fractions.

**Table 1:** Pharmacological activities reported in the literature for metabolites identified in the three fractions obtained from the methanolic extract of the root biomass of *T. parthenium* culture.

Compound	Pharmacological Activity				Reference
	Biological Effect	MIC	IC_50_	% Inhibition	
4-hydroxybenzoic acid	Antibacterial against *E. coli*	125 µg/mL			[59]
	Antibacterial against *S. aureus*	62.5 µg/mL			
	α-Amylase inhibition		3.552 mg/mL		[60]
	Antioxidant		321.72 µg/mL for DPPH		[59]
	Cytotoxic		20.8 µg/mL in MCF-7		
Behenic acid	Cytotoxic		7.52 µM in HepG2		[61]
			11.86 µM in MCF-7		
			12.28 µM in PC3		
	Antioxidant		˃1 mg/mL for DPPH		[62]
Chlorogenic acid	Antibacterial against *E. coli*	6 mg/mL			[63]
	Antibacterial against *S. aureus*	3 mg/mL			
	α-Amylase inhibition		9.1 µg/mL		[64]
	Antioxidant		51.23 µg/mL for DPPH		
	Cytotoxic			52% at 100 µM in 2OS	[65]
				27% at 100 µM in MG-63	
Gallic acid	Antibacterial against *E. coli*	1 µg/mL			[66]
	Antibacterial against *S. aureus*	1 µg/mL			
	α-Amylase inhibition		1.09 µg/mL		[67]
	Antioxidant		5.73 µM for DPPH		
	Cytotoxic		50 µM in SMMC-7721		[68]
			80 µM in HL-60		
			4 µM in k562		
			40 µM in Wehi231		
			80 µM in HeLa		
Gentisic acid	Antibacterial against *E. coli*	4 mg/mL			[69]
	Antibacterial against *S. aureus*	4.15 mg/mL			
	α-Amylase inhibition		2.07 mg/mL		[70]
	Antioxidant		7.6 µM for DPPH		[71]
	Cytotoxic		14 mM in HepG2		[69]
Isochlorogenic acid b	Antioxidant		EC_50_ 9.4 µg/mL for DPPH		[72]
Kaempferol-3-O-glucoside	Antibacterial against *E. coli*	1.25 µg/mL			[73]
	Antibacterial against *S. aureus*	0.625 µg/mL			
	Antioxidant	1.25 µg/mL for DPPH			
Lignoceric acid	Antibacterial against *E. coli*	˃1 mg/mL			[62]
	Antibacterial against *S. aureus*	˃1 mg/mL			
	Antioxidant	˃1 mg/mL for DPPH			
Oleic acid	Antibacterial against *E. coli*	0.512 mg/mL			
	Antibacterial against *S. aureus*	0.256 mg/mL			
	Antioxidant		0.5 mg/mL for DPPH		
Protocatechuic acid	Antibacterial against *E. coli*	2.5 mg/mL			[63]
	Antibacterial against *S. aureus*	0.45 mg/mL			[74]
	α-Amylase inhibition		1.12 µg/mL		[67]
	Antioxidant		8.29 µM for DPPH		
	Cytotoxic			55% at 8 µmol/L in MCF-7	[75]
				60% at 8 µmol/L in A549	
				45% at 8 µmol/L in HepG2	
				42% at 8 µmol/L in HeLa	
				65% at 8 µmol/L in LNCaP	
Quercetin-3-glucoside	Antioxidant		2.39 mM TEAC		[76]
	Cytotoxic			70.44% at 50 µg/mL in HeLa	[77]
Sodium diacetate	Antibacterial against *E. coli*	0.31% (*w*/*v*)			[78]
	Antibacterial against *S. aureus*	0.31% (*w*/*v*)			
Variabilin	Cytotoxic		87.74 µM in PC3		[58]
			38.08 µM in MCF-7		
			˃100 µM in HT-29		

DPPH, 2,2-Diphenyl-1-picrylhydrazyl (DPPH data shown in this table are related to the corresponding assay); EC_50_, half maximal effective concentration; IC_50_, half maximal inhibitory concentration; MIC, minimum inhibitory concentration; TEAC Trolox equivalent antioxidant capacity. The cancer cell lines described in this table are: A549 human lung cancer; C6 glioma; CaCo-2 human colorectal cancer; CAL27 epithelial cell line isolated from tongue; Calu-6 human lung cancer; CEM leukemia; H3255 lung cancer; HCT-116 human colorectal cancer; HeLa cervical; HepG2 hepatocellular carcinoma; HL-60 leukemia; HSC-2 oral squamous cell carcinoma; HSG human salivary gland adenocarcinoma; HT-29 colorectal cancer; k562 leukemia; L1210 leukemia; LNCaP human prostate cancer; MCF-7 breast cancer; MG-63 osteosarcoma; PC3 human prostate cancer; SMMC-7721 hepatoma; U2OS osteosarcoma; U937 leukemia; Wehi231 lymphoma.

### 3.4. Future Perspectives of the Findings

Medicinal plants are a source of natural compounds that possess diverse chemical structures and are important for the pharmaceutical industry to produce food supplements and cosmetics, among other products. Moreover, they are bioactive principles for complementary and alternative medicines; thus, the identification of key chemical biomarkers (active or purely analytical) and quality confirmation must be carried out [79]. Given that the 4TP fraction described in this work showed an outstanding abundance of fatty acids, it could be tested for applications in the food and cosmetic industries [80].

Since the 1960s, the industrial production of phytochemicals through plant tissue culture has been examined worldwide. Interest in commercial application has promoted extensive research on the production of different types of secondary metabolites as potential drugs. However, despite those efforts in research, only a few commercial applications have been achieved [81]. Thus, different strategies, such as screening productive cell lines, implementing cultures in bioreactors, eliciting secondary metabolism, hairy root culture, micropropagation, biotransformation, and metabolic engineering [81], have been developed to increase in vitro productivity and improve the efficiency of secondary metabolite production. In this work, plant tissue culture combined with analytical techniques related to untargeted and targeted metabolomics was demonstrated to represent an alternative source for researching cell lines that can produce several bioactive compounds that may be identified as key chemical biomarkers. Further research must be carried out on the fractions obtained from the methanolic extract of the root culture of *T. parthenium*, particularly for 4TP, because of the great pharmacological effects that were exerted, such as increasing the productivity and efficiency of producing the pharmacologically active metabolites, e.g., chlorogenic acid, since it was determined that this metabolite was detected at high concentration. Chlorogenic acid has been reported to be distributed among different species; some of the main sources of this compound are green coffee (7–8 g/100 g for *Coffea canephora* and 4–6 g/100 g for *C. arabica*) [82], apple (0.41–1.16 mg/g), prunes (1.3–3.9 g/100 g), tomato (21.30–240.16 µg/g), and carrot (0.3–18.8 mg/g) [82]; in this work, the 5TP fraction was 37.9 mg/g. This secondary metabolite can produce several biological effects, such as antioxidant, anti-inflammatory, liver and kidney protector, antibacterial, antitumor, nervous system protector, and sugar and lipid metabolism regulator effects. Due to its biological activities, it has received great attention, mainly as an antioxidant for medicinal and food purposes [82]. In addition, the concentration of isochlorogenic acid may also be high due to the correlations established between abundance and phytochemical and pharmacological effects for this compound (rt_m/z of 5.599_515.1196); its high positive correlations with TPC, TPAC, and IC_50_ and antibacterial effect against *E. coli* (0.97, 0.96, 0.96, and 0.85, respectively) and its moderate positive correlations with cytotoxic effect (0.62) and antibacterial effect against *S. aureus* (0.43) (Figure 3c). However, further research on the fractions obtained from the methanolic extract of the root culture of *T. parthenium* is needed to validate the putative identification of these compounds detected through untargeted metabolomics and to quantify them.

## 4. Materials and Methods

### 4.1. Root Biomass Production

Half-strength Murashige and Skoog culture media for the cultivation of suspended roots were prepared by adding 40 g/L D-glucose (Sigma-Aldrich, St. Louis, MO, USA), 150 mg/L ascorbic, 100 mg/L citric acid (Sigma-Aldrich, St. Louis, MO, USA), and 3 mg/L α-naphtalenacetic acid (Sigma-Aldrich, St. Louis, MO, USA) [36]. The culture medium pH was adjusted to 5.8 and then sterilized by autoclaving at 121 °C for 18 min. Roots (12 g fresh weight) were inoculated in a 1000 mL Erlenmeyer flask with 200 mL of liquid culture medium; the resulting root culture was placed in an orbital shaker at 110 rpm, and incubated at 25 ± 2 °C under white fluorescent light (50 µmol m^−2^ s^−1^) under a 16 h/8 h light/dark photoperiod for 32 days. The harvested biomass was filtered with a vacuum pump, rinsed with distilled water, and dried in an oven for 24 h at 60 °C.

### 4.2. Obtaining Fractions and Phytochemical Analysis

#### 4.2.1. Methanolic Extraction from Root Biomass

Dried root biomass (3 g) was extracted with 500 mL of methanol (MeOH) for 30 min in an ultrasonic homogenizer (Ningbo YinZhou Sjia Lab Equipment Co., LTD, model SJIA-950 W, Ningbo, China) configured at 20 kHz and 50 °C, operating for 9 s on and 6 s off. The extract was filtered using a vacuum pump and filter paper and then concentrated on a rotary evaporator (Büchi Labortechnik AG, R-300, Flawil, Switzerland) until dry.

#### 4.2.2. Fractionation of Methanolic Extract

Fractionation was performed on a chromatographic column employing silica gel (mesh size 60–200, Sigma-Aldrich, St. Louis, MO, USA) as the stationary phase, and the polarity was increased by mixing (A) acetonitrile and (B) MeOH. Ten different fractions were obtained with a volume of 100 mL each. An aliquot of every fraction (1 mL) was qualitatively analyzed with Baljet, ferric chloride, and sodium hydroxide tests to detect sesquiterpene lactones, phenolic compounds, and flavonoids, respectively [83]. The fractions with the most visually perceptible results (coded as 4TP, 5TP, and 8TP) were chosen for quantitative phytochemical analysis and for in vitro assays. The selected fractions (4TP, 5TP, and 8TP) were concentrated in a rotary evaporator (Büchi Labortechnik AG, R-300, Flawil, Switzerland) until almost dry and brought to dryness in an oven at 40 °C for 24 h.

### 4.3. In Vitro Assays of Selected Fractions

#### 4.3.1. Antioxidant Assay

The half maximal inhibitory concentration (IC_50_) of free radical scavenging was determined with 2,2-diphenyl-1-picrylhydrazyl (DPPH) radical to evaluate the antioxidant effect of the 4TP, 5TP, and 8TP fractions according to the methods of Nieto-Trujillo et al. [83]. A 0.1 mM methanolic solution of DPPH (Sigma-Aldrich, St. Louis, MO, USA) was prepared. Moreover, three drops of dimethyl sulfoxide (DMSO; Sigma-Aldrich, St. Louis, MO, USA) were added to each fraction, which was subsequently dissolved in MeOH to prepare different concentrations (0.1–10 mg/mL). The assay involved the combination of 1.8 mL of 0.1 mM DPPH with 0.3 mL of different concentrations of each fraction or MeOH (a blank control of the fractions). This mixture was incubated for 15 min in the dark at room temperature. Finally, the absorbance was measured at 515 nm in a UV-Vis spectrophotometer (Thermo Scientific, model Evolution 60S, Waltham, MA, USA). Every measurement was made in triplicate (n = 3). The results are expressed as the amount (in µg/mL) of the fraction capable of inhibiting the DPPH radical at 50% (IC_50_).

#### 4.3.2. Antibacterial Assay

The assay consisted of the Kirby–Bauer method and was tested via disk diffusion against *Staphylococcus aureus* (ATCC 25923) and *Escherichia coli* (ATCC 25922). For both strains, the bacterial inoculum was prepared in 5 mL of trypticase soy broth (Becton, Dickinson and Company, Sparks, MD, USA), adjusted to 0.5 on the McFarland scale to 1 × 10^6^ CFU/mL, and incubated at 37 °C for 24 h. Subsequently, 80 μL of inoculum was spread onto Müller–Hinton agar (Becton, Dickinson and Company, Sparks, MD, USA) plates using an L-shaped plastic rod, and sterile filter paper disks were placed. The fractions (4TP, 5TP, and 8TP) were dissolved in 3% (*v*/*v*) DMSO (Sigma-Aldrich, St. Louis, MO, USA) in MeOH to prepare solutions at concentrations of 100, 200, 400, 600 and 800 μg/mL; 10 µL of each solution was added per disk to achieve concentrations of 1, 2, 4, 6, and 8 μg/disk. Vancomycin (Sigma-Aldrich, St. Louis, MO, USA) and chloramphenicol (Sigma-Aldrich, St. Louis, MO, USA) (1 μg/disk) were used as positive inhibitory controls for *S. aureus* and *E. coli*, respectively, while 3% DMSO (Sigma-Aldrich, St. Louis, MO, USA) in MeOH was used as a blank control for the fractions, while sterile water was used as a negative control. The plates were incubated at 37 °C for 24 h, after which the inhibition halo was measured in mm using a Vernier caliper. This trial was performed with seven repeats per triplicate for each concentration against each bacterial strain (n = 21). The data are shown as a percentage of inhibition according to the following formula [83]:

$$\%\;inhibition=\frac{ZHI\;of\;fraction\;-\;ZHI\;of\;negative\;control}{ZHI\;of\;positive\;control\;-\;ZHI\;of\;negative\;control}\;*\;100$$

where ZHI corresponds to zone halo inhibition in mm.

#### 4.3.3. Cytotoxicity Assay

The human B non-Hodgkin lymphoma (Burkitt) cell line (Ramos RA-1) (ATCC, CRL-1596) was cultured in RPMI 1640 Advanced medium (Invitrogen^®^, Waltham, MA, USA) supplemented with 5% fetal bovine serum (FBS; Invitrogen^®^, Waltham, MA, USA), 1% antibiotic–antifungal mixture containing 10,000 U/mL penicillin G, 10 mg/mL streptomycin, and 25 μg/mL amphotericin B (Invitrogen^®^, Waltham, MA, USA). A total of 50,000 cells per well were cultured in 96-well plates with Advanced-RPMI 1640 medium and 2% FBS. The different fractions were dissolved in 0.01% (*v*/*v*) DMSO (Sigma-Aldrich, St. Louis, MO, USA) in culture media at final concentrations of 1, 5, 10, 20, 30, and 40 μg/mL, alone or in combination with 1 nM vincristine (Sigma-Aldrich, St. Louis, MO, USA). The controls for this assay consisted of 0.01% (*v*/*v*) DMSO in culture medium (negative control) and 1 nM vincristine (positive control). 50 μL of cells and 50 μL of fraction/control solution were inoculated into each well. The cell-inoculated plates were incubated for 24 h at 37 °C and 5% CO_2_. Treatments consisting of the combination of vincristine with the fraction, the vincristine solution was added 6 h before 24 h of incubation. Afterward, the exclusion staining method was used to count the dead and living cells. Thus, 20 μL was taken from each well of the plate, previously resuspended, and mixed with 80 μL of trypan blue (Invitrogen^®^, Waltham, MA, USA); then, 10 μL was placed on each side of a Neubauer chamber, and viable cells were counted on an inverted microscope (Olympus IX73, Evident Scientific, Inc., Waltham, MA, USA) with a 40× objective. The resulting data were used to estimate the cellular viability, expressed as a percentage of viable cells relative to total cells according to the following formula [83]:

$$Cellular\;viability\;(\%)=\frac{Number\;of\;total\;cells\;-\;Number\;of\;dead\;cells}{Number\;of\;total\;cells}\;*\;100$$

In addition, for each fraction, the results of this assay were used to determine the IC_50_ value using the equation for calculating the percentage of cell viability versus the concentration tested.

#### 4.3.4. α-Amylase Inhibitory Assay

The procedure consisted of testing the inhibitory effect of the selected fractions at different concentrations on α-amylase enzyme activity, according to Nieto-Trujillo et al. [83]. For this assay, 2 U of porcine α-amylase enzyme (Sigma-Aldrich, St. Louis, MO, USA) was dissolved in phosphate buffer solution (PBS); 0.5% starch (Sigma-Aldrich, St. Louis, MO, USA) was prepared in distilled water; and reactive dinitrosalicylic acid (DNSA; Sigma -Aldrich, St. Louis, MO, USA) was also used. The different fractions were dissolved in 0.01% (*v*/*v*) DMSO and distilled water to obtain 10 μg/mL solutions. Acarbose (Sigma-Aldrich, St. Louis, MO, USA) was used as a positive control, and distilled water was used as a negative control. The absorbance was measured at 540 nm in a UV-Vis spectrophotometer (Thermo Scientific, model evolution 60S, Waltham, MA, USA) using water as a blank. The absorbance results are expressed as a percentage of inhibition according to the following formula [83]:

$$\%Inhibition=\frac{b-a}{b}\;*\;100$$

where:

a=Absorbance T1 − (Absorbance T2 − Absorbance T3)

b=Absorbance T4−Absorbance T5

The treatments related to variables coded T1 to T5 are shown in Table S4.

In addition, different water solutions of acarbose (0.1, 0.65, 1.25, 2.5, 3.5 and 5 μg/mL) were prepared to construct a calibration curve (y = 15.141x + 12.316, R^2^ = 0.9408), from which the IC_50_ was estimated. This calibration curve was used to express the results of the α-amylase inhibition as mg of acarbose equivalents per gram of fraction (mg AE/gF) [83].

### 4.4. Phytochemical Analysis of the Selected Fractions

#### Total Secondary Metabolite Contents

The total contents of phenolic compounds, phenolic acids, flavonoids, and sesquiterpene lactones (TPC, TPAC, TFC, and TSLC, respectively) were determined according to the methods of Nieto-Trujillo et al. [83] using gallic acid, quercetin, and verbascoside standards (Sigma-Aldrich, St. Louis, MO, USA), respectively, to construct the calibration curves. The results are expressed as milligrams of gallic acid, quercetin, or verbascoside equivalents per gram of fraction (mg GAE/gF, mg QE/gF, or mg VBE/gF), respectively. TSLC was conducted according to Salapovic et al. [84] using PTN as a standard (Sigma-Aldrich, St. Louis, MO, USA). The TSLC results are expressed as milligrams of PTN equivalents per gram of fraction (mg PTNE/gF). The absorbance measurements were carried out in triplicate (n = 3) using a UV-Vis spectrophotometer (Thermo Scientific, model evolution 60S, Waltham, MA, USA).

### 4.5. Metabolomics of Fractions

#### 4.5.1. Untargeted Metabolomics

The 4TP, 5TP, and 8TP fractions were analyzed via liquid chromatography coupled with mass spectrometry (LC-MS) to perform untargeted metabolomics. The LC-MS system consisted of a 1290 infinity Agilent ultrahigh resolution liquid chromatograph (UPLC) coupled to a 6545 Agilent quadrupole time-of-flight mass spectrometer (Q-TOF/MS) (Agilent Technologies, Santa Clara, CA, USA). Chromatographic separations were performed on an ACQUITY UPLC BEH C18 column (1.7 µm, 2.1 × 50 mm; Agilent Technologies, Santa Clara, CA, USA). The mobile phase consisted of (A) water with 0.1% formic acid and (B) MeOH with 0.1% formic acid, both of which were of mass spectrometry grade. The gradient elution profile was as follows: 90% in the first 2 min A, 2–10 min 90–40% A, 10–15 min 40% A, 15–20 min 40–10%, 20–25 min 10% A, 25–27 min 10–90% A, and 27–31 min 90% A. The flow rate was set to 0.4 mL/min. The column temperature was 40 °C. The injection volume was 10 µL. All samples were analyzed with three replicates. The mass spectrometer used a dual AJS ESI ion source. The ESI source was operated in negative and positive mode with a gas temperature of 300 °C, cone gas (N_2_) flow rate of 10 L/min, nebulizer pressure of 20 psi, sheath gas temperature of 300 °C, sheath gas flow rate of 10 L/min, and skimmer offset of 65 V with a fragmentor of 120 V. The mass spectrum data were acquired with a mass range from 100 to 3000 *m*/*z*. Multiple mass spectrophotometric scanning modes, including full scanning, product ion scanning, and positive/negative scanning were used for qualitative analysis. MS/MS experiments were performed to determine fragmentation patterns by means of the acquisition mode untargeted MS and a fixed collision energy of 15 eV. During this analytical study, quality control samples (seven reference masses) were analyzed to assess the variance observed in the acquired data.

Chromatographic data were processed using MassHunter Qualitative Analysis Software (version B.08.00; Agilent Technologies, Santa Clara, CA, USA). The Molecular Feature Extraction algorithm extracted compounds from the raw data. The MetaboAnalyst bioinformatic platform (https://www.metaboanalyst.ca/, accessed on 20 August 2024) was used to analyze the untargeted data via the statistical analysis module; a principal component analysis (PCA), a partial least squares discriminant analysis (PLS-DA), a variable importance in projection (VIP), and a hierarchical clustering heatmap were generated [85]. In addition, the data obtained through untargeted metabolomics analysis (rt_*m*/*z* signals and their abundances based on peak intensities) were correlated with the results of the total secondary metabolite contents of the fractions and the in vitro assays by testing a Pearson correlation; those *m*/*z* signals correlated with a pharmacological effect were putatively identified with the FooDB Version 1.0 database (https://foodb.ca/, accessed 15 October 2024). The mass spectra of those putatively identified compounds are shown in the Supplementary Material (Figure S1).

#### 4.5.2. Targeted Metabolomics

The confirmatory identification of selected phenolic compounds obtained through untargeted metabolomics was performed using targeted metabolomics. This procedure was performed according to the procedure reported by Monribot-Villanueva et al. [86]. Briefly, each fraction was dissolved in MeOH with 0.1% formic acid at 50 mg/mL, filtered through 0.2 µm PTFE membranes and placed in 2 mL UPLC vials. The samples were analyzed in a 1290 infinity Agilent UPLC coupled to a 6460 Agilent triple quadrupole mass spectrometer (Agilent Technologies, Santa Clara, CA, USA). Chromatographic separations were performed on an ECLIPSE PLUS C18 column (1.8 µm, 2.1 × 50 mm; Agilent Technologies, Santa Clara, CA, USA). The mobile phase consisted of (A) water with 0.1% formic acid and (B) acetonitrile with 0.1% formic acid, both of which were MS grade. The gradient elution profile, regarding the mobile phase A, was as follows: 99–50% in the first 30 min, 30–35 min 50–1%, 35–39 min 1%, 39–40 min 1–99%, and 40–45 min 99%. The flow rate was set to 0.3 mL/min. The column temperature was 40 °C. The injection volume was 2 µL. All the samples were analyzed with three replicates. The mass spectrometer was operated at a gas temperature of 300 °C, gas flow rate of 5 L/min, nebulizer pressure of 45 psi, sheath gas temperature of 250 °C, sheath gas flow rate of 11 L/min, capillary voltage (positive and negative) of 3500 V and nozzle voltage (positive and negative) of 500 V. The analytical conditions for identifying and quantifying phenolic compounds are shown in Table S5. The concentrations of the identified compounds are expressed in µg/g of the dry fraction. In addition, the MetaboAnalyst bioinformatic platform (https://www.metaboanalyts.ca/, accessed on 20 November 2024) was used for building a heatmap through a statistical analysis module to compare the concentrations of all the quantified phenolic compounds among the three fractions.

### 4.6. Statistical Analysis

The total secondary metabolite contents (TPC, TPAC, TFC, and TSLC) and in vitro assay results (IC_50_ for the antioxidant assay, percentage inhibition of bacteria and α-amylase, and percentage of viability in RA-1) were statistically analyzed through ANOVA, followed by a multiple media/median comparison test. NCSS software (2007 version) was used for all the ANOVA and Pearson’s correlation analyses, and R 4.4.2 software was used for obtaining heatmaps; in all the statistical analyses, a *p* < 0.05 was assumed to indicate significant differences.

## 5. Conclusions

The root culture of *T. parthenium* is a potential source of several metabolites, such as phenolic acids, fatty acids, coumarins, sesquiterpenoids, and triterpenoids, which are capable of exerting α-amylase inhibition and antioxidant, antibacterial, and cytotoxic effects. Because of the pharmacological effects of these fractions, further research must be carried out to estimate their functionality as a source of bioactive principles in several applications, such as the development of drugs and the formulation of cosmetics and nutraceuticals, among others. The potential of plant tissue culture as a source of several pharmacologically active compounds could be estimated by in vitro bioassays combined with analytical techniques such as untargeted and targeted metabolomics, which might enhance the current limitation in establishing commercial applications of plant tissue culture because it is aimed at producing key bioactive compounds.

## Data Availability

The data that support the findings of this study are available from the corresponding author on request.

## Supplementary Materials

The following supporting information can be downloaded at: https://www.mdpi.com/article/10.3390/ijms26157209/s1.

## Abbreviations

The following abbreviations are used in this manuscript:

4TP, 5TP or 8TP	Fractions obtained from the methanolic extract of the root biomass of the *T. parthenium* culture
AJS	Agilent jet stream
ATCC	American Type Culture Collection
CFU	Colony-forming units
DMSO	Dimethyl sulfoxide
DNSA	Dinitrosalicylic acid
DPPH	2,2-Diphenyl-1-picrylhydrazyl
EC_50_	Half maximal effective concentration
ESI	Electrospray ionization
FBS	Fetal bovine serum
IC_50_	Half maximal inhibitory concentration
LC-MS	Liquid chromatography coupled with mass spectrometry
MeOH	Methanol
mg AE/gF	Milligrams of acarbose equivalents per gram of fraction
mg GAE/gF	Milligrams of gallic acid equivalents per gram of fraction
mg QE/gF	Milligrams of quercetin equivalents per gram of fraction
mg VBE/gF	Milligrams of verbascoside equivalents per gram of fraction
mg PTNE/gF	Milligrams of parthenolide equivalents per gram of fraction
MIC	Minimum inhibitory concentration
MS	Mass spectrometry
*m*/*z*	Mass charge signal
PBS	Phosphate buffer solution
PCA	Principal component analysis
PGR	Plant growth regulators
PLS-DA	Partial least squares discriminant analysis
PTN	Parthenolide
Q-TOF/MS	Quadrupole time-of-flight mass spectrometer
rt	Retention time in minutes
TFC	Total flavonoids content
TPAC	Total phenolic acid content
TPC	Total phenolic compound content
TSLC	Total sesquiterpene lactone content
UPLC	Ultrahigh resolution liquid chromatograph
VIP	Variable importance in projection
ZHI	Zone halo inhibition
