# Supplementary material for: Metabolomic and Pharmacological Approaches for Exploring the Potential of Tanacetum parthenium L. Root Culture as a Source of Bioactive Phytochemicals
Source: Int J Mol Sci. 2025 Jul 25;26(15):7209. doi: 10.3390/ijms26157209 (PMC12346442; doi:10.3390/ijms26157209)
Supplement: Supplementary file 1 [file ijms-26-07209-s001.zip › ijms-3740530-Supplementary Materials.pdf]

(a)

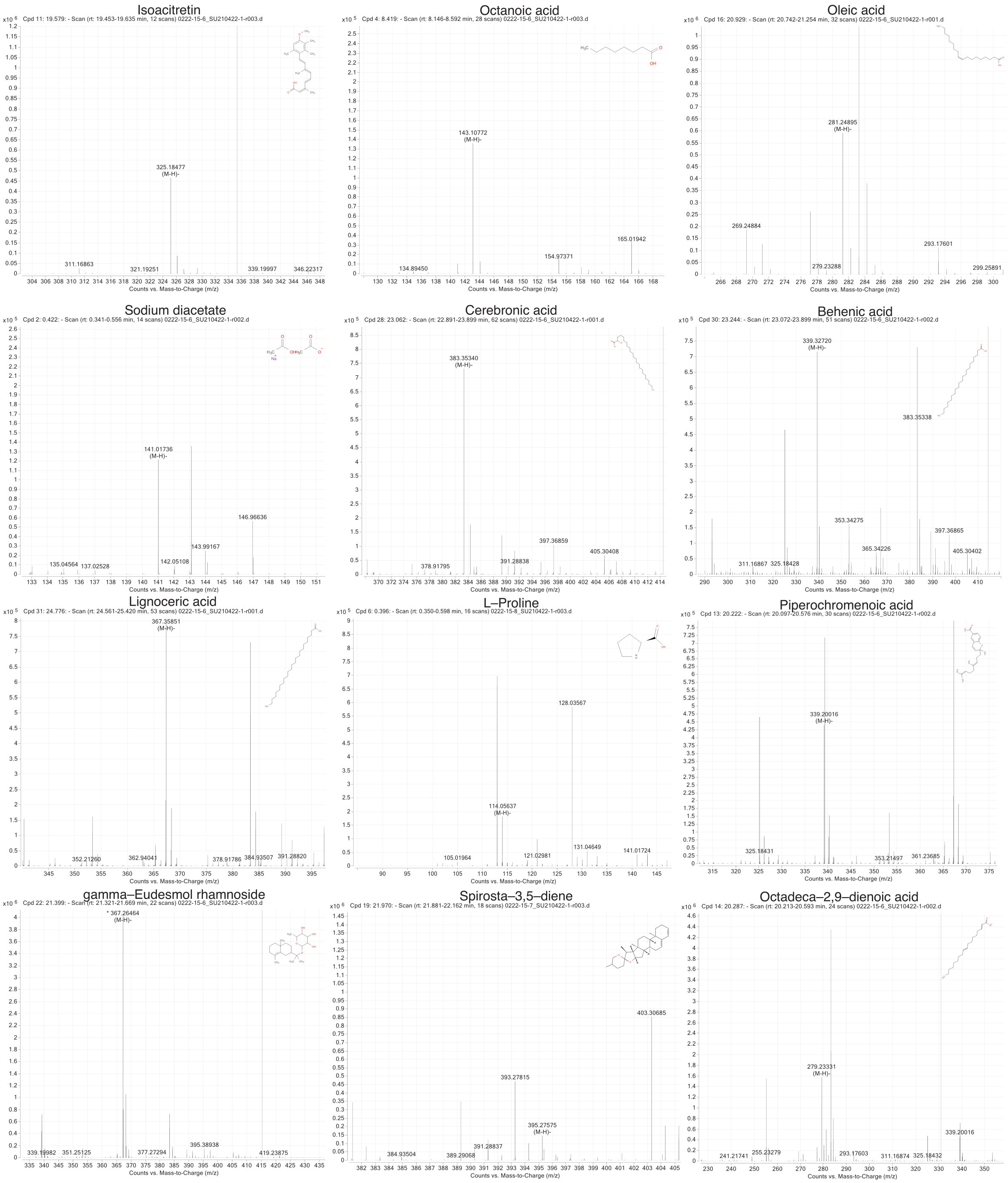
Isoacitretin
Cpd 11: 19.579: - Scan (rt: 19.453-19.635 min, 12 scans) 0222-15-6_SU210422-1-r003.d
325.18477 (M-H)-
311.16863
321.19251
339.19997
346.22317
Counts vs. Mass-to-Charge (m/z)
Octanoic acid
Cpd 4: 8.419: - Scan (rt: 8.146-8.592 min, 28 scans) 0222-15-6_SU210422-1-r003.d
143.10772 (M-H)-
134.89450
154.97371
165.01942
Counts vs. Mass-to-Charge (m/z)
Oleic acid
Cpd 16: 20.929: - Scan (rt: 20.742-21.254 min, 32 scans) 0222-15-6_SU210422-1-r001.d
281.24895 (M-H)-
269.24884
279.23288
293.17601
299.25891
Counts vs. Mass-to-Charge (m/z)
Sodium diacetate
Cpd 2: 0.422: - Scan (rt: 0.341-0.556 min, 14 scans) 0222-15-6_SU210422-1-r002.d
141.01736 (M-H)-
146.96636
143.99167
142.05108
135.04564
137.02528
Counts vs. Mass-to-Charge (m/z)
Cerebronic acid
Cpd 28: 23.062: - Scan (rt: 22.891-23.899 min, 62 scans) 0222-15-6_SU210422-1-r001.d
383.35340 (M-H)-
397.36859
405.30408
391.28838
378.91795
Counts vs. Mass-to-Charge (m/z)
Behenic acid
Cpd 30: 23.244: - Scan (rt: 23.072-23.899 min, 51 scans) 0222-15-6_SU210422-1-r002.d
339.32720 (M-H)-
383.35338
353.34275
397.36865
365.34226
405.30402
311.16867
325.18428
Counts vs. Mass-to-Charge (m/z)
Lignoceric acid
Cpd 31: 24.776: - Scan (rt: 24.561-25.420 min, 53 scans) 0222-15-6_SU210422-1-r001.d
367.35851 (M-H)-
352.21260
362.94041
378.91786
384.93507
391.28820
Counts vs. Mass-to-Charge (m/z)
L–Proline
Cpd 6: 0.396: - Scan (rt: 0.350-0.598 min, 16 scans) 0222-15-8_SU210422-1-r003.d
128.03567
114.05637 (M-H)-
105.01964
121.02981
131.04649
141.01724
Counts vs. Mass-to-Charge (m/z)
Piperochromenoic acid
Cpd 13: 20.222: - Scan (rt: 20.097-20.576 min, 30 scans) 0222-15-6_SU210422-1-r002.d
339.20016 (M-H)-
325.18431
353.21497
361.23685
Counts vs. Mass-to-Charge (m/z)
gamma–Eudesmol rhamnoside
Cpd 22: 21.399: - Scan (rt: 21.321-21.669 min, 22 scans) 0222-15-6_SU210422-1-r003.d
* 367.26464 (M-H)-
339.19982
351.25125
377.27294
395.38938
419.23875
Counts vs. Mass-to-Charge (m/z)
Spirosta–3,5–diene
Cpd 19: 21.970: - Scan (rt: 21.881-22.162 min, 18 scans) 0222-15-7_SU210422-1-r003.d
403.30685
393.27815
395.27575 (M-H)-
384.93504
389.29068
391.28837
Counts vs. Mass-to-Charge (m/z)
Octadeca–2,9–dienoic acid
Cpd 14: 20.287: - Scan (rt: 20.213-20.593 min, 24 scans) 0222-15-6_SU210422-1-r002.d
279.23331 (M-H)-
339.20016
241.21741
255.23279
293.17603
311.16874
325.18432
Counts vs. Mass-to-Charge (m/z)

(b)

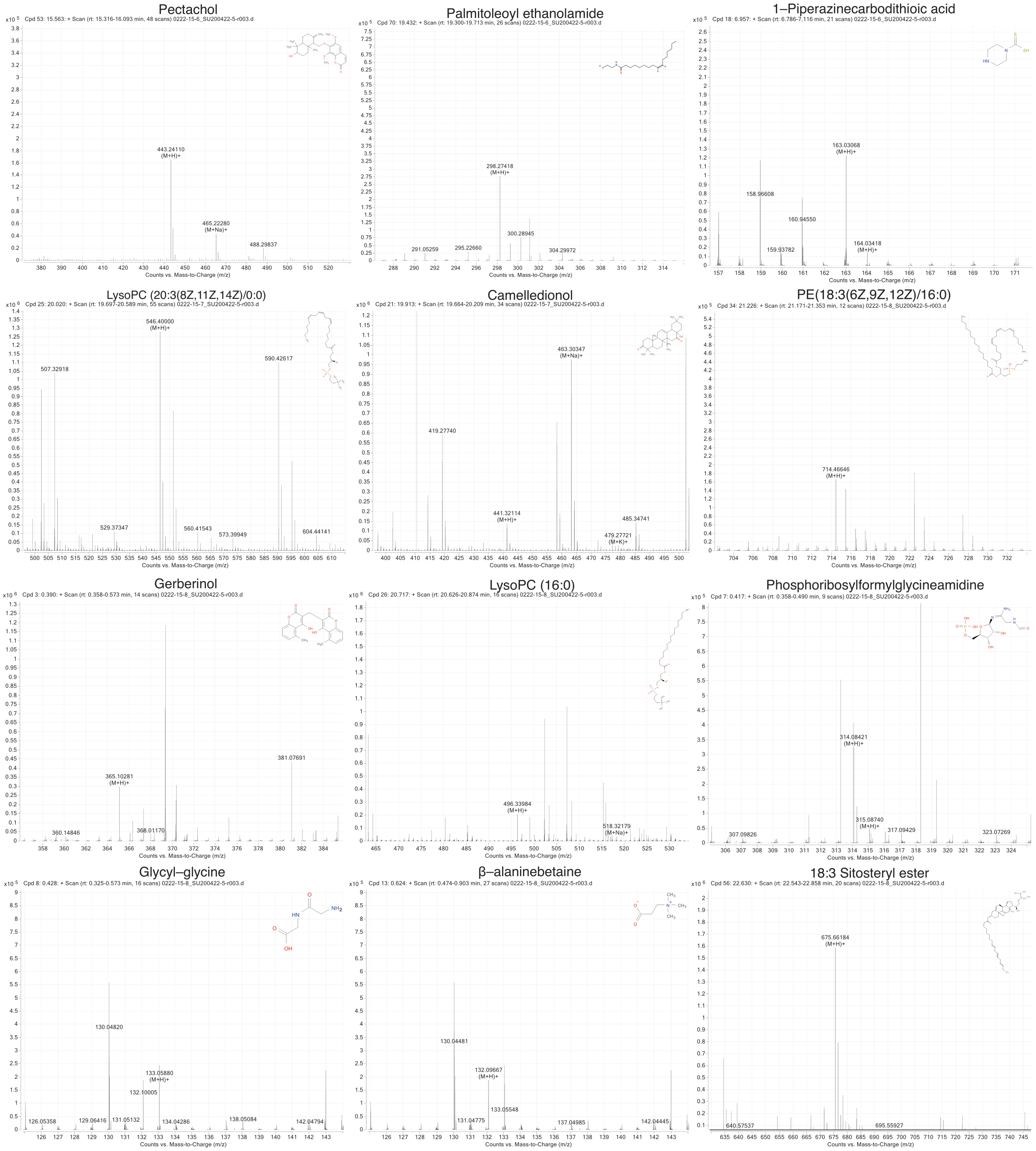

Pectachol
Cpd 53: 15.563: + Scan (rt: 15.316-16.093 min, 48 scans) 0222-15-6_SU200422-5-r003.d
443.24110 (M+H)+
465.22280 (M+Na)+
488.29837
Palmitoleoyl ethanolamide
Cpd 70: 19.432: + Scan (rt: 19.300-19.713 min, 26 scans) 0222-15-6_SU200422-5-r003.d
298.27418 (M+H)+
291.05259
295.22660
300.28945
304.29972
1–Piperazinecarbodithioic acid
Cpd 18: 6.957: + Scan (rt: 6.786-7.116 min, 21 scans) 0222-15-6_SU200422-5-r003.d
163.03068 (M+H)+
158.96608
160.94550
159.93782
164.03418 (M+H)+
LysoPC (20:3(8Z,11Z,14Z)/0:0)
Cpd 25: 20.020: + Scan (rt: 19.697-20.589 min, 55 scans) 0222-15-7_SU200422-5-r003.d
546.40000 (M+H)+
507.32918
590.42617
529.37347
560.41543
573.39949
604.44141
Camelledionol
Cpd 21: 19.913: + Scan (rt: 19.664-20.209 min, 34 scans) 0222-15-7_SU200422-5-r003.d
463.30347 (M+Na)+
419.27740
441.32114 (M+H)+
485.34741
479.27721 (M+K)+
PE(18:3(6Z,9Z,12Z)/16:0)
Cpd 34: 21.226: + Scan (rt: 21.171-21.353 min, 12 scans) 0222-15-8_SU200422-5-r003.d
714.46646 (M+H)+
Gerberinol
Cpd 3: 0.390: + Scan (rt: 0.358-0.573 min, 14 scans) 0222-15-8_SU200422-5-r003.d
365.10281 (M+H)+
381.07691
360.14846
368.01170
LysoPC (16:0)
Cpd 26: 20.717: + Scan (rt: 20.626-20.874 min, 16 scans) 0222-15-8_SU200422-5-r003.d
496.33984 (M+H)+
518.32179 (M+Na)+
Phosphoribosylformylglycineamidine
Cpd 7: 0.417: + Scan (rt: 0.358-0.490 min, 9 scans) 0222-15-8_SU200422-5-r003.d
314.08421 (M+H)+
307.09826
315.08740 (M+H)+
317.09429
323.07269
Glycyl–glycine
Cpd 8: 0.428: + Scan (rt: 0.325-0.573 min, 16 scans) 0222-15-8_SU200422-5-r003.d
130.04820
133.05880 (M+H)+
132.10005
126.05358
129.06416
131.05132
134.04286
138.05084
142.04794
β–alaninebetaine
Cpd 13: 0.624: + Scan (rt: 0.474-0.903 min, 27 scans) 0222-15-8_SU200422-5-r003.d
130.04481
132.09667 (M+H)+
133.05548
131.04775
137.04985
142.04445
18:3 Sitosteryl ester
Cpd 56: 22.630: + Scan (rt: 22.543-22.858 min, 20 scans) 0222-15-8_SU200422-5-r003.d
675.66184 (M+H)+
640.57537
695.55927
Counts vs. Mass-to-Charge (m/z)

(c)

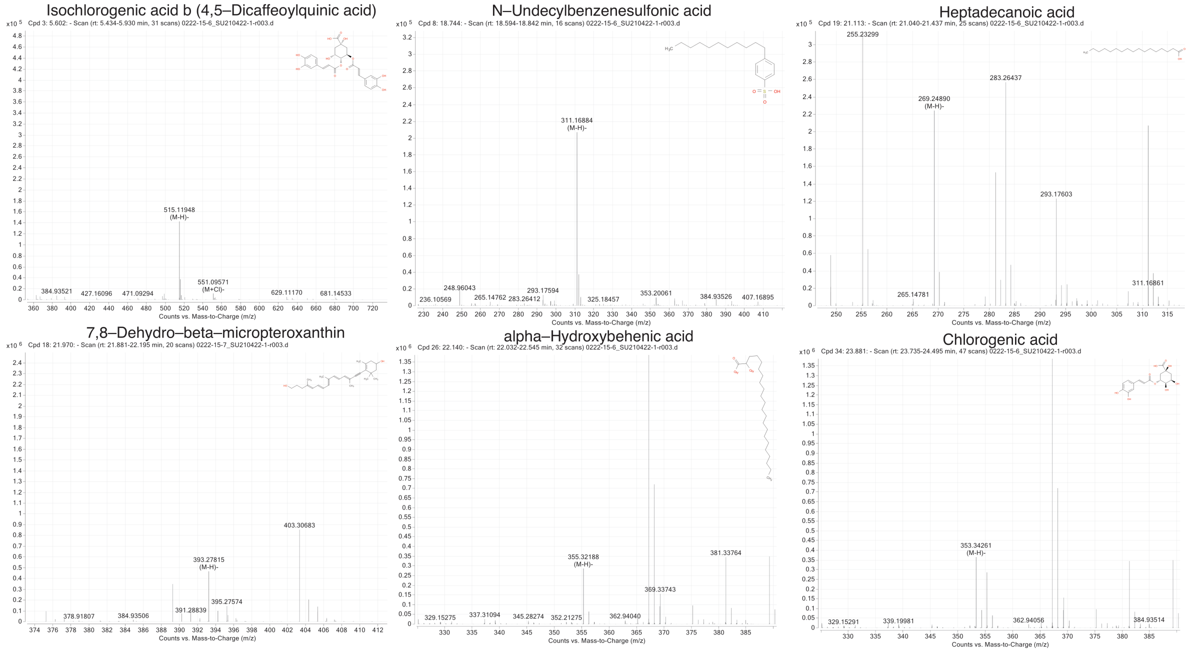

(d)

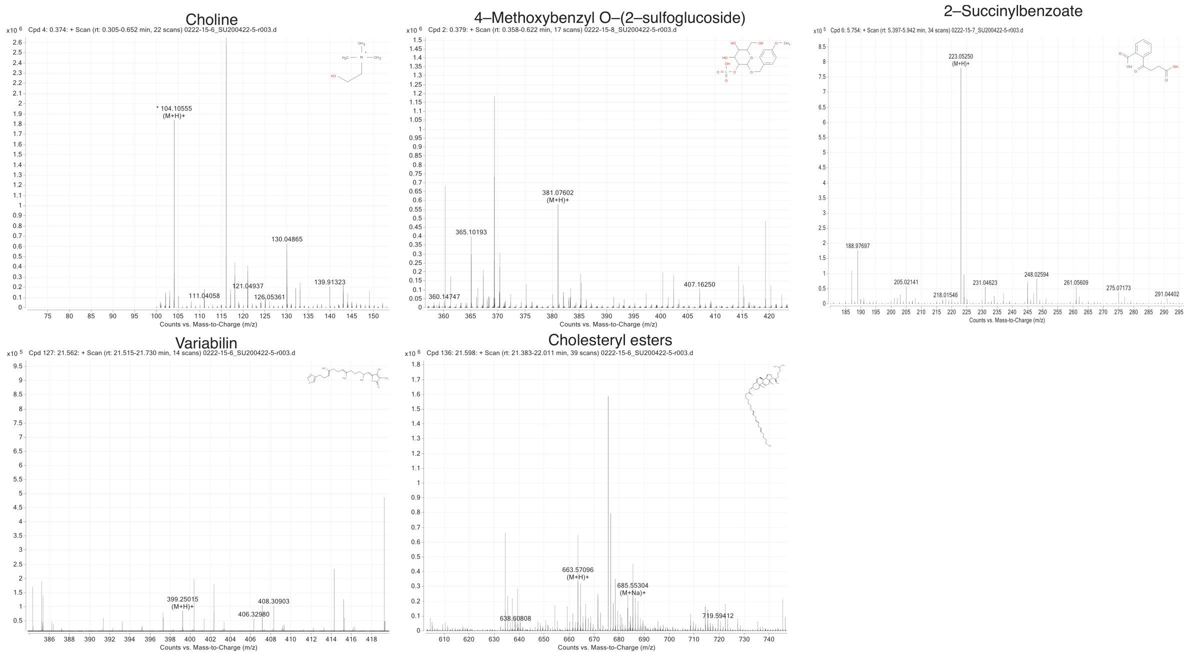

Figure S1. Full-scan mass spectrum ion chromatograms, in electrospray negative and positive ion mode, of metabolites related to pharmacological effects of 4TP, 5TP, and 8TP fractions, obtained from a methanolic extract of root biomass of *T. parthenium* culture. Metabolites related to pharmacological effects in (a) negative and (b) positive ion mode; metabolites related to VIP score in (c) negative and (d) positive ion mode. The mass spectra were obtained from Agilent MassHunter Qualitative Analysis B.07.00. Images of the chemical structures were obtained from the PubChem database

(https://pubchem.ncbi.nlm.nih.gov/, accessed 15 October 2024). The putative identification was made in FooDB Version 1.0 database (https://foodb.ca/, accessed 15 October 2024).

Table S1: Putative identification of metabolites correlated with pharmacological effects determined in fractions.

Data results from mass spectrometry analysis				Correlation between compound abundance and pharmacological effect			Putative identification				
Retention time (min)	m/z	Ion mode	Adduct type	Fraction	Pharmacological effect	Correlation	Name	Class of compounds	Formula	Monoisotopic mass	Mass error (ppm)
6.960	163.0305	positive	M+H	4TP	Amylase inhibition	0.43135	1-Piperazinecarbodithioic acid	Piperazine	$C_5H_{10}N_2S_2$	162.0285	33
8.419	143.1078	negative	M-H	4TP	Antioxidant	0.30764	Octanoic acid	Saturated fatty acid	$C_8H_{16}O_2$	144.115	0
15.557	443.2387	positive	M+H	4TP	Antibacterial vs *E. coli*	0.4584	Pectachol	Coumarins and derivatives	$C_{26}H_{34}O_6$	442.2355	9
19.432	298.2733	positive	M+H	4TP	Antibacterial vs *E. coli*	0.42258	Palmitoleoyl ethanolamide	n-Acylethanolamines/fatty amide	$C_{18}H_{35}NO_2$	297.2668	3
19.432	298.2733	positive	M+H	4TP	Amylase inhibition	0.39221	Palmitoleoyl ethanolamide	n-Acylethanolamines/fatty amide	$C_{18}H_{35}NO_2$	297.2668	3
19.432	298.2733	positive	M+H	4TP	Antioxidant	0.30156	Palmitoleoyl ethanolamide	n-Acylethanolamines/fatty amide	$C_{18}H_{35}NO_2$	297.2668	3
19.587	325.1846	negative	M-H	4TP	Antioxidant	0.37167	Isoacitretin	Retinoids	$C_{21}H_{26}O_3$	326.1882	11
19.963	463.3025	positive	M+Na	4TP	Antibacterial vs *S. aureus*	0.38972	Camelledionol	Triterpenoid	$C_{27}H_{42}O_6$	462.2981	34
20.938	281.2489	negative	M-H	4TP	Antioxidant	0.3863	Oleic acid	Monounsaturated omega-9 fatty acid	$C_{18}H_{34}O_2$	282.2559	1
0.422	141.0175	negative	M-H	5TP	Amylase inhibition	0.29915	Sodium diacetate	Acetate salts	$C_4H_7NaO_4$	142.0242	4
0.422	141.0175	negative	M-H	5TP	Antibacterial vs *E. coli*	0.54309	Sodium diacetate	Acetate salts	$C_4H_7NaO_4$	142.0242	4
0.422	141.0175	negative	M-H	5TP	Antibacterial vs *S. aureus*	0.51163	Sodium diacetate	Acetate salts	$C_4H_7NaO_4$	142.0242	4
19.963	463.3025	positive	M+Na	5TP	Cytotoxic	0.66336	Camelledionol	Triterpenoid	$C_{27}H_{42}O_6$	462.2981	34
19.963	463.3025	positive	M+Na	5TP	Amylase inhibition	0.33049	Camelledionol	Triterpenoid	$C_{27}H_{42}O_6$	462.2981	34
19.963	463.3025	positive	M+Na	5TP	Antibacterial vs *E. coli*	0.32708	Camelledionol	Triterpenoid	$C_{27}H_{42}O_6$	462.2981	34
20.020	546.4003	positive	M+H	5TP	Cytotoxic	0.40772	LysoPC(20:3(8Z,11Z,14Z)/0:0)	Lysophosphatidylethanolamines	$C_{28}H_{52}NO_7P$	545.3481	82
20.020	546.4003	positive	M+H	5TP	Antibacterial vs *S. aureus*	0.37924	LysoPC(20:3(8Z,11Z,14Z)/0:0)	Lysophosphatidylethanolamines	$C_{28}H_{52}NO_7P$	545.3481	82

23.059	383.3532	negative	M-H	5TP	Antioxidant	0.20193	Cerebronic acid	Long-chain fatty acids	$C_{24}H_{48}O_3$	384.3603	0
23.244	339.3269	negative	M-H	5TP	Antioxidant	0.41239	Behenic acid	Long-chain fatty acids	$C_{22}H_{44}O_2$	340.3341	0
23.244	339.3269	negative	M-H	5TP	Antibacterial vs *E. coli*	0.26424	Behenic acid	Long-chain fatty acids	$C_{22}H_{44}O_2$	340.3341	0
23.244	339.3269	negative	M-H	5TP	Amylase inhibition	0.17984	Behenic acid	Long-chain fatty acids	$C_{22}H_{44}O_2$	340.3341	0
24.774	367.3583	negative	M-H	5TP	Antibacterial vs *S. aureus*	0.33479	Lignoceric acid	Long-chain fatty acids	$C_{24}H_{48}O_2$	368.3654	0
24.774	367.3583	negative	M-H	5TP	Antibacterial vs *E. coli*	0.28548	Lignoceric acid	Long-chain fatty acids	$C_{24}H_{48}O_2$	368.3654	0
0.393	365.0973	positive	M+H	8TP	Cytotoxic	0.42091	Gerberinol	4-Hydroxycoumarins	$C_{21}H_{16}O_6$	364.0947	13
0.393	365.0973	positive	M+H	8TP	Antioxidant	0.33253	Gerberinol	4-Hydroxycoumarins	$C_{21}H_{16}O_6$	364.0947	13
0.397	114.0565	negative	M-H	8TP	Antioxidant	0.43417	L-Proline	Amino acid	$C_5H_9NO_2$	115.0633	4
0.397	114.0565	negative	M-H	8TP	Antibacterial vs *E. coli*	0.4273	L-Proline	Amino acid	$C_5H_9NO_2$	115.0633	4
0.421	314.0813	positive	M+H	8TP	Cytotoxic	0.12821	Phosphoribosylformylglycineamidine	Pentose phosphate	$C_8H_{16}N_3O_8P$	313.0675	21
0.430	133.0585	positive	M+H	8TP	Antibacterial vs *S. aureus*	0.43317	Glycyl-glycine	Simplest peptide, made of two glycine molecules	$C_4H_8N_2O_3$	132.0535	10
0.430	133.0585	positive	M+H	8TP	Cytotoxic	0.38148	Glycyl-glycine	Simplest peptide, made of two glycine molecules	$C_4H_8N_2O_3$	132.0535	10
0.624	132.0964	positive	M+H	8TP	Amylase inhibition	0.31307	β-alanine betaine	Tetraalkylammonium salts	$C_6H_{13}NO_2$	131.0946	42
19.587	325.1846	negative	M-H	8TP	Amylase inhibition	0.19925	Isoacitretin	Retinoids	$C_{21}H_{26}O_3$	326.1882	11
20.215	339.2001	negative	M-H	8TP	Antibacterial vs *S. aureus*	0.42885	Piperochromenoic acid	Bicyclic monoterpenoids	$C_{22}H_{28}O_3$	340.2038	10
20.287	279.2332	negative	M-H	8TP	Amylase inhibition	0.2312	Octadeca-2,9-dienoic acid	Lineolic acids and derivatives	$C_{18}H_{32}O_2$	280.2402	1
20.716	496.3399	positive	M+H	8TP	Antibacterial vs *S. aureus*	0.51656	LysoPC (16:0)	Lysophosphatidylcholine(18:2/0:0)	$C_{24}H_{50}NO_7P$	495.3325	0
21.224	714.4634	positive	M+H	8TP	Antibacterial vs *E. coli*	0.30497	PE(18:3(6Z,9Z,12Z)/16:0)	Cholesterol ester	$C_{39}H_{72}NO_8P$	713.4996	61
21.224	714.4634	positive	M+H	8TP	Cytotoxic	0.30002	PE(18:3(6Z,9Z,12Z)/16:0)	Cholesterol ester	$C_{39}H_{72}NO_8P$	713.4996	61
21.224	714.4634	positive	M+H	8TP	Antioxidant	0.23604	PE(18:3(6Z,9Z,12Z)/16:0)	Cholesterol ester	$C_{39}H_{72}NO_8P$	713.4996	61

21.227	367.2446	negative	M-H	8TP	Amylase inhibition	0.41788	gamma-Eudesmol rhamnoside	Eudesmane sesquiterpenoid	$C_{21}H_{36}O_5$	368.2563	12
21.227	367.2446	negative	M-H	8TP	Cytotoxic	0.12361	gamma-Eudesmol rhamnoside	Eudesmane sesquiterpenoid	$C_{21}H_{36}O_5$	368.2563	12
21.955	395.2759	negative	M-H	8TP	Antibacterial vs *S. aureus*	0.493	Spirosta-3,5-diene	Triterpenoid	$C_{27}H_{40}O_2$	396.3028	50
22.630	675.6476	positive	M+H	8TP	Cytotoxic	0.17447	Sitosteryl-18:3	Triterpenoid	$C_{47}H_{78}O_2$	674.6002	59

The putative identification was made in FooDB Version 1.0 database (https://foodb.ca/, accessed 15 October 2024).

Table S2: Putative identification of main compounds determined in the VIP score

Data results from mass spectrometry analysis				VIP score	Putative identification				
Retention time (min)	m/z	Ion mode	Adduct type		Name	Class of compounds	Formula	Monoisotopic mass	Mass error (ppm)
0.374	104.1067	positive	M+H	1.4934	Choline	N, N, N-trimethylethanolammonium cation	$C_5H_{14}NO$	104.1075	8
0.380	381.0676	positive	M+H	1.8475	4-Methoxybenzyl O-(2-sulfoglucoside)	o-Glycosyl compounds	$C_{14}H_{20}O_{10}S$	380.0777	46
0.422	141.0175	negative	M-H	1.0172	Sodium diacetate	Acetate salt	$C_4H_7NaO_4$	142.0242	4
5.599	515.1196	negative	M-H	1.0208	Isochlorogenic acid b	Quinic acids and derivatives	$C_{25}H_{24}O_{12}$	516.4509	82
5.752	223.0517	positive	M+H	1.4113	2-Succinylbenzoate	Alkyl-phenylketones.	$C_{11}H_{10}O_5$	222.0528	38
6.960	163.0305	positive	M+H	1.0584	1-Piperazinecarbodithioic acid	Piperazine	$C_5H_{10}N_2S_2$	162.0285	33
18.744	311.1689	negative	M-H	1.1003	N-Undecylbenzenesulfonic acid	Benzenesulfonic acids and derivatives	$C_{17}H_{28}O_3S$	312.1759	1
20.215	339.2001	negative	M-H	1.0789	Piperochromenoic acid	Bicyclic monoterpenoids	$C_{22}H_{28}O_3$	340.2038	10
21.126	269.2489	negative	M-H	1.0288	Heptadecanoic acid	Long-chain fatty acids	$C_{17}H_{34}O_2$	270.2559	1
21.567	399.2503	positive	M+H	1.0498	Variabilin	Diterpenoids	$C_{25}H_{34}O_4$	398.2457	7
21.609	663.5713	positive	M+H	1.0284	Cholesteryl esters	Cholesteryl esters	$C_{45}H_{74}O_3$	662.5638	0

21.955	395.2759	negative	M-H	1.0327	Spirosta-3,5-diene	Triterpenoid	$C_{27}H_{40}O_2$	396.3028	50
21.970	393.2783	negative	M-H	1.0128	7,8-Dehydro-beta-micropteroxanthin	Sesquiterpenoid	$C_{27}H_{38}O_2$	394.2872	4
22.158	355.3219	negative	M-H	1.0299	alpha-Hydroxybehenic acid	Long-chain fatty acids	$C_{22}H_{44}O_3$	356.329	0
23.059	383.3532	negative	M-H	1.8905	Cerebronic acid	Long-chain fatty acids	$C_{24}H_{48}O_3$	384.3603	0
23.224	339.3269	negative	M-H	2.0373	Behnic acid	Long-chain fatty acids	$C_{22}H_{44}O_2$	340.3341	0
23.916	353.3427	negative	M-H	1.0228	Chlorogenic acid	Quinic acids and derivatives	$C_{16}H_{18}O_9$	354.3087	95
24.774	367.3583	negative	M-H	1.8995	Lignoceric acid	Long-chain fatty acids	$C_{24}H_{48}O_2$	368.3654	0

The putative identification was made in FooDB Version 1.0 database (https://foodb.ca/, accessed 15 October 2024).

Table S3: Concentrations of the phenolic compounds and L-phenylalanine detected in the 4TP, 5TP, and 8TP fractions obtained from the methanolic extract of the root biomass of *T. parthenium* culture.

**Compound**	**Concentration (μg/gF) of the compound in the fraction**		
	**4TP**	**5TP**	**8TP**
Aromatic amino acid			
Phenylalanine	3.22 ± 0.07	22.64 ± 0.51	119.14 ± 4.41
Hydroxybenzoic acids			
4-Hydroxybenzoic acid	3.70 ± 0.04	4.98 ± 0.06	ND
Gallic acid	1.84 ± 0.01	3.91 ± 0.01	1.04 ± 0.07
Gentisic acid	2.67 ± 0.05	4.69 ± 0.08	ND
Protocatechuic acid	2.23 ± 0.03	6.39 ± 0.02	2.27 ± 0.05
Caffeoylquinic acids			
Chlorogenic acid	4204.70 ± 83.20	37902.80 ± 341.19	4119.20 ± 195.15
Flavonoids			
Quercetin-3-glucoside	6.19 ± 0.39	7.25 ± 0.24	ND
Kaempferol-3-O-glucoside	2.09 ± 0.14	7.80 ± 0.10	1.59 ± 0.13

The data are presented as the means ± SDs. The concentration for each compound is expressed in μg/g of the dried fraction. ND: Compounds not detected.

Table S4. Variables codes related to modifications of the procedure used to determine the inhibition of the α-amylase enzyme catalytic activity.

Variable code	Enzyme	PBS buffer	Water	Starch	DNSA	Sample
T1	√	√				√
T2	√	√			√	√
T3	√		√	√		√
T4	√		√	√	√	
T5	√	√	√		√	

Table S5: Analytical conditions for identification and quantification of phenolic compounds.

Compound	dMRM transition			Mass spectrometric conditions			Quantification conditions		
	Precursor ion	Product ion	Retention time	Collision energy	Fragmentor	Polarity	Quantification range (µM)	Regression type	R^2
Shikimic acid[1]	173.1	111.1	0.53	10	100	Negative	0.5 - 19	Quadratic	0.99
Gallic acid[2]	169.0	125.2	1.56	10	100	Negative	0.5 - 19	Quadratic	0.99
L-Phenylalanine[3]	166.1	131.0	2.16	10	100	Positive	0.5 - 19	Quadratic	0.99
Protocatechuic acid[1]	153.0	109.1	2.91	10	100	Negative	0.5 - 19	Quadratic	0.99
4-Hydroxybenzoic acid[1]	137.1	92.8	4.38	10	100	Negative	0.5 - 19	Quadratic	0.99
Gentisic acid[1]	153.0	109.0	4.44	10	100	Negative	0.5 - 19	Quadratic	0.99
Vainilloside[2]	315.1	153.0	4.94	10	100	Positive	0.5 - 19	Quadratic	0.99
4-Hydroxyphenylacetic acid[3]	107.1	77.0	5.38	20	140	Positive	0.5 - 19	Quadratic	0.99
(-)-Epigallocatechin[4]	305.1	125.0	5.44	20	140	Negative	0.5 - 19	Quadratic	0.99
(+)-Catechin[2]	291.0	138.9	5.73	10	100	Positive	0.5 - 19	Quadratic	0.99
Vanillic acid[1]	169.0	93.0	5.93	10	100	Positive	0.5 - 19	Quadratic	0.99
Chlorogenic acid[1]	355.1	163.0	6.14	10	100	Positive	0.5 - 19	Quadratic	0.99
Caffeic acid[1]	181.0	163.0	6.15	10	100	Positive	0.5 - 19	Quadratic	0.99
Scopolin[5]	355.1	193.0	6.18	20	100	Positive	0.5 - 19	Quadratic	0.99
Malvin[1]	655.1	331.1	6.42	40	100	Positive	0.5 - 19	Quadratic	0.99
Kuromanin[1]	449.0	286.9	6.98	30	100	Positive	0.5 - 19	Quadratic	0.99
Procyanidin B2[1]	577.1	425.1	7.24	10	100	Negative	0.5 - 19	Quadratic	0.99
Keracyanin[2]	595.2	287.1	7.56	20	100	Positive	0.5 - 19	Quadratic	0.99
Vanillin[1]	153.0	124.9	7.65	10	100	Positive	0.5 - 19	Quadratic	0.99
(-)-Epicatechin[2]	291.0	138.8	7.68	10	100	Positive	0.5 - 19	Quadratic	0.99
Mangiferin[2]	423.0	302.8	8.00	10	100	Positive	0.5 - 19	Quadratic	0.99

4-Coumaric acid[1]	165.0	147.0	8.23	10	100	Positive	0.5 - 19	Quadratic	0.99
(-)-Gallocatechin gallate[2]	458.9	139.0	8.74	20	80	Positive	0.5 - 19	Quadratic	0.99
Umbelliferone[1]	163.0	107.0	8.81	30	100	Positive	0.5 - 19	Quadratic	0.99
Quercetin 3,4-di-O-glucoside[1]	627.0	302.9	9.51	10	100	Positive	0.5 - 19	Quadratic	0.99
Scopoletin[1]	193.0	133.0	9.63	10	100	Positive	0.5 - 19	Quadratic	0.99
Ferulic acid[1]	195.1	145.0	9.69	20	100	Positive	0.5 - 19	Quadratic	0.99
3-Coumaric acid[1]	165.05	147.04	9.78	10	100	Positive	0.5 - 19	Quadratic	0.99
Sinapic acid[1]	225.1	207.1	10.26	10	100	Positive	0.5 - 19	Quadratic	0.99
Salicylic acid[2]	137.0	93	10.70	10	100	Negative	0.5 - 19	Quadratic	0.99
Epicatechin gallate[4]	443.1	123.0	10.71	10	100	Positive	0.5 - 19	Quadratic	0.99
Ellagic acid[1]	300.5	145.0	10.72	30	170	Negative	0.5 - 19	Quadratic	0.99
Myricitrin[1]	465.0	318.9	10.82	10	100	Positive	0.5 - 19	Quadratic	0.99
Quercetin 3-D-galactoside[2]	465.0	302.9	11.09	10	100	Positive	0.5 - 19	Quadratic	0.99
Rutin[2]	611.0	302.9	11.12	10	100	Positive	0.5 - 19	Quadratic	0.99
Quercetin 3-glucoside[2]	465.0	303.0	11.38	10	100	Positive	0.5 - 19	Quadratic	0.99
Luteolin 7-O-glucoside[1]	449.0	287.0	11.60	10	100	Positive	0.5 - 19	Quadratic	0.99
p-Anisic acid[3]	153.1	109.0	11.68	5	120	Positive	0.5 - 19	Quadratic	0.99
Penta-O-galloyl-B-D-glucose[2]	771.1	153.0	12.45	20	100	Positive	0.5 - 19	Quadratic	0.99
2,4-Dimethoxy-6-methylbenzoic acid[2]	197.0	179.0	12.57	5	80	Positive	0.5 - 19	Quadratic	0.99
Kaemperol 3-O-glucoside[1]	449.0	286.9	12.76	10	100	Positive	0.5 - 19	Quadratic	0.99
Quercitrin[1]	449.1	303.1	12.79	10	100	Positive	0.5 - 19	Quadratic	0.99
Naringin[1]	273.0	153.0	12.91	10	120	Positive	0.5 - 19	Quadratic	0.99
Myricetin[1]	317.0	179.0	13.16	10	100	Negative	0.5 - 19	Quadratic	0.99
Hesperidin[1]	609.1	301.1	13.48	20	100	Negative	0.5 - 19	Quadratic	0.99
Rosmarinic acid[1]	361.1	163.0	13.62	10	100	Positive	0.5 - 19	Quadratic	0.99

trans-Resveratrol[2]	229.1	135.0	13.81	10	100	Positive	0.5 - 19	Quadratic	0.99
Phloridzin[1]	435.0	272.9	13.84	10	100	Negative	0.5 - 19	Quadratic	0.99
Secoisolariciresinol[2]	363.2	137.1	13.98	20	100	Positive	0.5 - 19	Quadratic	0.99
trans-Cinnamic acid[1]	149.1	131.0	15.31	10	100	Positive	0.5 - 19	Quadratic	0.99
Quercetin[2]	301	151	16.17	20	100	Negative	0.5 - 19	Quadratic	0.99
Luteolin[1]	285	151	16.33	20	100	Negative	0.5 - 19	Quadratic	0.99
Psoralen[1]	187.0	131.1	16.82	20	100	Positive	0.5 - 19	Quadratic	0.99
Angelicin[2]	187.0	131.1	17.49	20	100	Positive	0.5 - 19	Quadratic	0.99
Naringenin[1]	271.0	151	17.97	10	100	Negative	0.5 - 19	Quadratic	0.99
Apigenin[1]	271.0	153.0	18.68	30	100	Positive	0.5 - 19	Quadratic	0.99
Kaempferol[1]	285	151	18.99	10	100	Negative	0.5 - 19	Quadratic	0.99
Hesperetin[1]	303.1	177.1	19.26	20	100	Positive	0.5 - 19	Quadratic	0.99
Podophyllotoxin[4]	415.1	397.1	20.18	10	100	Positive	0.5 - 19	Quadratic	0.99
Methyl cinnamate[2]	163.1	131.0	23.21	6	100	Positive	0.5 - 19	Quadratic	0.99
Nordihydroguaiaretic acid[1]	303.0	193.1	23.73	10	100	Positive	0.5 - 19	Quadratic	0.99
Chrysin[1]	255.1	153.0	24.37	40	100	Positive	0.5 - 19	Quadratic	0.99
Kaempferide[1]	299	284	25.75	20	100	Positive	0.5 - 19	Quadratic	0.99
Emodin[1]	269.0	225.0	28.79	20	150	Negative	0.5 - 19	Quadratic	0.99
Chrysophanol[1]	255.1	153.0	32.47	40	100	Positive	0.5 - 19	Quadratic	0.99

The retention time variation allowed for the search of the compounds was 2 min in each case. The cell accelerator voltage was 7 V for each compound. Dilutions were made if the concentration of some compounds was higher than the linearity range. Compounds were purchased from [1] Extrasynthese, Lyon, Genay, France; [2] Sigma-Aldrich, St. Louis, MO, USA; [3] kindly donated by Dr. Thor Arnason (University of Ottawa); [4] Cayman Chemical Company, Ann Arbor, Michigan, USA; [5] isolated in-house.
